# Whole-Genome Sequencing and Comparative Genome Analysis of *Bacillus subtilis* Strains Isolated from Non-Salted Fermented Soybean Foods

**DOI:** 10.1371/journal.pone.0141369

**Published:** 2015-10-27

**Authors:** Mayumi Kamada, Sumitaka Hase, Kazushi Fujii, Masato Miyake, Kengo Sato, Keitarou Kimura, Yasubumi Sakakibara

**Affiliations:** 1 Department of Biosciences and Informatics, Keio University, 3-14-1 Hiyoshi, Kohoku-ku, Yokohama 223-8522, Japan; 2 Department of Biological Sciences, The University of Tokyo, 2-11-16 Yayoi, Bunkyo-ku, Tokyo 113-0032, Japan; 3 Division of Applied Microbiology, National Food Research Institute, 2-1-12 12 Kannondai, Tsukuba, Ibaraki 305-8642, Japan; University of California Davis, UNITED STATES

## Abstract

*Bacillus subtilis* is the main component in the fermentation of soybeans. To investigate the genetics of the soybean-fermenting *B. subtilis* strains and its relationship with the productivity of extracellular poly-*γ*-glutamic acid (*γ*PGA), we sequenced the whole genome of eight *B. subtilis* stains isolated from non-salted fermented soybean foods in Southeast Asia. Assembled nucleotide sequences were compared with those of a natto (fermented soybean food) starter strain *B. subtilis* BEST195 and the laboratory standard strain *B. subtilis* 168 that is incapable of *γ*PGA production. Detected variants were investigated in terms of insertion sequences, biotin synthesis, production of subtilisin NAT, and regulatory genes for *γ*PGA synthesis, which were related to fermentation process. Comparing genome sequences, we found that the strains that produce *γ*PGA have a deletion in a protein that constitutes the flagellar basal body, and this deletion was not found in the non-producing strains. We further identified diversity in variants of the *bio* operon, which is responsible for the biotin auxotrophism of the natto starter strains. Phylogenetic analysis using multilocus sequencing typing revealed that the *B. subtilis* strains isolated from the non-salted fermented soybeans were not clustered together, while the natto-fermenting strains were tightly clustered; this analysis also suggested that the strain isolated from “Tua Nao” of Thailand traces a different evolutionary process from other strains.

## Introduction

Microbial organisms have a huge influence on the environment and human health. Especially, fermentation by microorganisms plays an important role in food processing, not only in the preservation of foods but also the biological enrichment of food substrates with vitamins, protein, essential amino acids, and essential fatty acids, thus increasing the nutritional value [1]. Moreover, fermentation also enhances the health-promoting effects of soybeans. Fermented soybeans contain significantly more isoflavone genistein than non-fermented soybeans [2], and it has been reported as a chemopreventive agent against cancer [3]. *Bacillus subtilis* is the main component in the alkaline fermentation of soybeans without salt, which is common in East and South-east Asia and in West Africa as a seasoning or side dishes. *B. subtilis* is the best-characterized gram-positive bacterium and often is used as a model organism. About 30 groups from the USA, Japan, Korea, and Europe sequenced and annotated the whole genome of the laboratory standard strain *B. subtilis* subsp. *subtilis* 168 [4]. *B. subtilis* (natto) strain BEST195 whose genome was sequenced by third-generation sequencing technology [5] is a closely related organism of *B. subtilis* 168 and produces “natto”, which is a non-salted fermented soybean food that is mainly consumed in Japan.

In the process of natto production, *B. subtilis* (natto) synthesizes some useful products for human health and industry, such as poly-*γ*-glutamic acid (*γ*PGA), which is the major constituent of viscous material and is a useful polymer for biomedical and industrial applications [6, 7]. Actually, *γ*PGA has been used to purify water in some developing countries [8], and the experimental study using mice reported that *γ*PGA is effective against atopic dermatitis that is a chronic inflammatory skin disease [9]. On the other hand, *B. subtilis* 168 is incapable of the production of *γ*PGA. However, not every *B. subtilis* that ferment soybeans does not produce *γ*PGA, and vice versa; that is, a favorable phenotype for fermentation cannot be predicted only by the production of *γ*PGA [10]. Therefore, unraveling the genetics of *B. subtilis* strains that can be used in the production of non-salted fermented foods is of high interest, and it would be helpful for the efficient production of useful material produced in the fermentation process.

In Southeast Asia, there are some non-salted fermented soybean foods similar to natto, including “Chungkuk Jang” in Korea, “Kinema” in Nepal, “Tua Nao” in northern Thailand, “Pepoke” in Myanmar and “Mac Tua Nao” in northern Laos. These foods are made in the same way as natto, but after fermentation, they are made into a paste. There are many ways to eat, some of fermented soybean pastes are placed in the sun to make them like cracker, and then used as seasoning. To characterize *B. subtilis* strains that are used to produce fermented soybean foods, ninety *B. subtilis* strains have been isolated from fermented soybean foods, including the above foods, and molecular biological investigations have been performed in terms of biotin requirement, productivity of *γ*PGA protease and amylase, phage type and inheritance of insertion sequence (IS) [11]. The IS appeared to be widely distributed among *B. subtilis* strains isolated from non-salted types of fermented soybeans and a relatively small fraction of *B. subtilis* from salted types of fermented soybeans. IS elements are considered to be related to genetic competence of *B. subtilis* and genetic instability of *γ*PGA production in BEST195 [12, 13]. However, no relationship between IS element and *γ*PGA production was apparent in the experimental study [11], and the relevance of IS presence to natto fermentation is still unknown.

To understand the genetics and diversity of the strains producing non-salted fermented soybean foods in more depth, we sequenced the whole genomes of eight strains isolated from non-salted fermented soybean foods in six countries (Korea, Myanmar, Nepal, Thailand, Laos, and Japan) and performed comparative genome analyses with *B. subtilis* subsp. *subtilis* BEST195, which is a starter strain used for natto production and has been sequenced completely in our previous work [5]. In this paper, we describe the assembly and the annotation of the genomes of eight strains and variant analysis focusing on some important features of the soybean-fermenting strains. We also performed phylogenetic analysis using multilocus sequence typing (MLST). Using the assembled genome sequences and identified nucleotide changes from the BEST195 genome, we found that differences in biotin synthesis and a nucleotide deletion in a flagella motor protein potentially affected the production of *γ*PGA. Additionally, the MLST results indicated that the non-salted soybean-fermenting strains were not classified as a single group.

## Materials and Methods

### Bacterial strains and genomic DNA extraction

*B. subtilis* strains used in this study and source materials where they were isolated were listed in Table 1. Strains, Miyagino, Takahashi, and Naruse are three major natto starter strains used in Japan. The BEST195 strain whose genome was used as a reference genome is the Miyagino strain. Strains NARUSE and TAKAHASHI were purchased from Naruse Fermentation Chemical Laboratory K.K. (Tokyo, Japan) and Yuzo Takahashi Laboratory (Yamagata, Japan), respectively. The strain NAFM5 is a derivative of Miyagino whose plasmids were removed [14]. Five strains isolated from the non-salted fermented soybean foods of Asia were used: strain KorC1 was isolated from “Chungkuk Jang” in Korea, strain LaoA1 was isolated from “Mac Tua Nao” in Laos, strain MyaA2 was isolated from “Chine Pepoke” in Myanmar, strain ThaB was isolated from “Tua Nao” in Thailand, and strain NepD5 was isolated from “Kinema” in Nepal. These five *B. subtilis* strains were isolated as described previously [11]. Three of these five strains and a strain (LaoA3) isolated from the same source as LaoA1 were tested in terms of the production ability of *γ*PGA in the previous study [11], and these results were listed in Table 1. Genomic DNA of *B. subtilis* was isolated from Luria Broth culture according to a routine biochemical isolation procedure [15].

**Table 1:** The detail of fermented soybean foods used as source and phenotypic characters of each strain.

Sample (original strain name in [11])	Fermented soybeans	Appearance	Country	*γ*PGA production in [11]
KorC1 (NFRI8338)	Chungkuk Jang	Raw paste	Korea	untested
LaoA1 (NFRI8302)	Mac Tua Nao	Raw paste	Laos	untested (LaoA3 is No)
MyaA2 (NFRI8316)	Chine Pepoke	Semi-dried block	Myanmar	No
ThaB (NFRI8347)	Tua Nao	Sun-dried chips	Thailand	Yes
NepD5 (NFRI8292)	Kinema	Sun-dried block	Nepal	No
NAFM5	Natto	Raw paste	Japan	untested
NARUSE	Natto	Raw paste	Japan	untested
TAKAHASHI	Natto	Raw paste	Japan	untested

LaoA3 is a strain isolated from the same source as LaoA1.

We used *B. subtilis* (natto) BEST195 (GenBank accession number AP011541.2) [5] as the reference genome. For comparison analysis and phylogenetic analysis, some relative strains were used, and their genomes were accessed from the following GenBank accession numbers: *B. subitlis* subsp. *subtilis* 168 (NC 000964) [16], *B. subtilis* subsp. *spizizenii* W23 (CP002183) [17], and *B. amyloliquefaciens* LL3 (CP002634) [18].

### Sequencing

Whole-genome shotgun sequencing was individually performed on the eight *B. subtilis* strains KorC1, LaoA1, MyaA2, ThaB, NepD5, NAFM5, NARUSE, and TAKAHASHI. Libraries for all strains were prepared using Paired-End Sample Prep Kit and Multiplexing Sample Preparation Oligonucleotide Kit (Illumina Inc., San Diego, CA, USA). DNA was sheared with a Covaris instrument (Covaris Inc., Woburn, MA, USA) to 500 bases, and fragmented DNAs were checked by an Agilent Bioanalyzer DNA 7500 kit. DNA fragments were enriched using a 10-cycle PCR for strains NARUSE and TAKAHASHI, and an 18-cycle PCR for the other strains. The amplified libraries were sequenced on a Genome Analyzer IIx (Illumina Inc., San Diego, CA, USA) instrument, generating 56-bp paired-end reads for strains NepD5, NAFM5, NARUSE, and TAKAHASHI and 58-bp paired-end reads for the other strains. To use only high-quality reads, short reads with a Phred quality below Q30 were filtered out using the FASTX-Toolkit (http://hannonlab.cshl.edu/fastx_toolkit/). Additionally, to remove Illlumina-specific sequencing errors, we applied Trowel [19], which is a *k*-mer spectrum-based error correction method for Illumina reads, with default parameters.

### Reference-assisted genome assembly and annotation

To obtain an accurate assembly and utilize the reference genome sequence, we performed the following three steps to assemble genomes. First, short reads were assembled using the *de novo* assembly tool SPAdes version 3.1.0 [20] with *k*-mer = (15, 21, 25, 31, 35, 39, 45). Assembled contigs were then aligned to the reference genome, and consensus sequences were extracted as super-contigs using AMOScmp package [21]. Finally, super-contigs were connected using SSPACE version 3.0 [22], which is a stand-alone scaffolder of pre-assembled contigs using paired-read data. The connected super-contigs were used as draft genomes for each strain. Assembly results were evaluated on the basis of N50, maximum contig length, and the number of contigs was calculated with QUality ASsesment Tool (QUAST) version 2.2 [23]. The gene prediction program Glimmer version 3.02 [24] for the prokaryote genome was applied to each draft genome with trans_table = 11. tRNAs were annotated with tRNAscan-SE version 1.3.1 [25], and rRNA was annotated using RNAmmer version 1.2 [26].

### Comparison of assembled genomes with *B. subtilis* BEST195 and 168

To identify orthologous genes to *B. subtilis* BEST195 and 168, we used the reciprocal best hit (RBH) method with BLASTx [27]. In this method, a gene *i* in species *A* is an RBH of gene *j* in species *B* if a query of species *A* with gene *i* yields gene *j* as the top hit with more than 80% identity, and a reciprocal query of species *B* with gene *j* yields gene *i* as the top hit with more than 80% identity. Here, we accounted for the alignment length of each gene and checked whether or not a gene *i*(*j*) is aligned to gene *j*(*i*) with more than 80% of its sequence length. We aligned each assembled draft genome to the reference genome (BEST195) using NUCmer, delta-filter and show-coords, which are modules in MUMmer version 3.23 [28], allowing local alignment. An image of aligned regions was generated using an original script and DNAplotter version 10.2 [29]. Note that from the above image, we can see only the result of local alignment with binary expression whether or not scaffolds in the draft genomes are aligned to the BEST195 genome.

### Analysis of insertion sequence (IS)

For each strain, we identified genes corresponding to transposases of the ISs, that is, IS*4Bsu1*, IS*Bma2*, IS*643*, IS*256Bsu1*, IS*Lmo1*, and putative transposase via BLASTx against BEST195 with more than 80% identity. The scaffolds of the draft genomes containing identified IS genes were aligned to the BEST195 genome using MUMmer. To investigate and illustrate positions of a transposase of IS against the BEST195 genome, the scaffolds of the draft genomes were ordered and oriented using ABACAS [30]. After eliminating scaffolds that did not align to the BEST195 genome, the ordered and oriented scaffolds were concatenated into a sequence and aligned with the BEST195 genome using Murasaki [31].

### Mapping and variants call

The short reads from each strain were aligned to the BEST195 genome using Burrows-Wheeler alignment (BWA) tool version 0.7.10 [32]. We sorted mapped reads and removed unmapped reads using SAMtools version 0.1.18 [33] and also removed duplicated reads using Picard tools (version 1.119; http://broadinstitute.github.io/picard/). Indel realignment and SNP/INDEL detection were then performed on each strain separately using the Genome Analysis Toolkit (GATK) version 3.2-2 [34] with the parameters -ploidy 1 and -glm BOTH to use a model for identifying SNPs and INDELs at the same time in a non-diploid organism. The detected variants were filtered by VariantFilteration in GATK with the following filter expressions: “DP < 10 ∣∣ QUAL < 100.0” and “QD < 5.0”.

Impacts for each detected variant were predicted at four levels, high, moderate, low, and modifier, using SNP effect predictor (SnpEff) version 3.4 [35]. First, we focused on variants predicted as high-, moderate-, and low-impact effects. High impact indicates that a variant is assumed to have a disruptive impact on the protein, such as frame shift, loss of start codon, and gain of stop codon. Moderate impact indicates a non-disruptive variant that might change the protein effectiveness, such as non-synonymous coding and codon change/insertion/deletion. Low impact indicates that a variant is assumed to be mostly harmless or unlikely to change protein behavior, such as a synonymous variation.

To analyze detected variants statistically, we calculated a genetic variant score for each gene based on the variant data with predicted impact effects. The genetic variant score of a gene *i* for a strain X is defined by

$${\text{gene}}_{i}^{\left(X\right)}=\log\left(C_{high}^{\left(X\right)}N_{high_{i}}^{\left(X\right)}+C_{mod}^{\left(X\right)}N_{mod_{i}}^{\left(X\right)}+C_{low}^{\left(X\right)}N_{low_{i}}^{\left(X\right)}+1\right),$$

where *N*_*high*_*i*__, *N*_*mod*_*i*__, and *N*_*low*_*i*__ are the number of variants in gene *i* annotated as high-, moderate- and low-effect impact, respectively, and *C* is a positive constant for each effect defined as follows:

$$C_{low}=1,C_{mod}=MAX_{low}/2,C_{high}=\left(C_{mod}+MAX_{mod}\right)/2,$$

where *MAX*_*low*_ and *MAX*_*mod*_ are the maximum number of low and moderate effects in each strain.

Two statistical analyses using the genetic variant vector, a principal component analysis (PCA), and hierarchical clustering based on the Euclidean distance and furthest neighbor methods were performed with R statistical package. For PCA, we did not normalize the variant matrices to norm 0 and variance 1 before performing PCA because the scores that calculated the above equation are comparable and no further preprocessing was necessary. For a detailed genetic analysis, we listed genes with variants with high and moderate impacts. Gene Ontology (GO) annotations for BEST195 genes were performed using Blast2GO version 3.0.7 [36] with a local BLASTx search against the nr database. The GO terms for listed genes with variants were counted and summarized at level 2 using Bioconductor R packages [37]. The level is defined on the GO graph in goProfiles, which is an R package for the statistical analysis of function profiles. Level 1 corresponds to three categories of GO terms: biological process, molecular function, and cellar component, and level 2 is a group of their directly connected children. For the variant analysis focusing on important genes, genome sequences for each strain were obtained by substituting the BEST195 genome sequence based on the detected variants using a script. Multiple sequence alignments and visualizations of the results were performed with CLC Sequence Viewer 7.5 (CLC Inc., Aarhus, Denmark). Plots of variant positions were generated using Gnuplot (version 4.4; http://gnuplot.sourceforge.net/).

### MLST analysis

MLST characterizes bacterial isolates on the basis of sequence polymorphism within internal fragments of seven housekeeping genes. In this study, MLST based on the sequences of internal fragments of the *glpF*, *ilvD*, *pta*, *purH*, *pycA*, *rpoD*, and *tpiA* genes was carried out on strains KorC1, LaoA1, MyaA2, ThaB, NepD5, NAFM5, NARUSE, TAKAHASHI, *B. subtilis* BEST195, 168, W23, and *B. amyloliquefaciens* LL3. The MLST sequences of strains KorC1, LaoA1, MyaA2, ThaB, NepD5, NARUSE, TAKAHASHI, BEST195, and 168 were identified using the *B. subtilis* MLST website (PubMLST; http://pubmlst.org/bsubtilis/) developed by Keith Jolley and sited at the University of Oxford [38], and those of other strains were obtained from this database. The obtained sequences were concatenated into one sequence for each strain using a script. The alignment of these sequences was performed with ClustalW using the Molecular Evolutionary Genetic Analysis (MEGA) 6.0 software [39]. The genetic distance between the sequences was calculated using the Kimura 2-parameter model [40], and the phylogenetic tree was constructed using the neighbor-joining algorithm with MEGA 6.0 software. Branch quality was assessed by the bootstrap test using 1500 replicates. *B. subtilis* W23 and *B. amyloliquefaciens* LL3 strains were used as the outgroups.

### *De novo* assembly with unmapped reads

As a result of mapping, some reads were left unmapped to the reference genome. These reads can be regarded as fragments of an extrachromosomal DNA such as a plasmid or differences from the reference genome. Thus, we extracted unmapped reads of each strain using SAMtools and assembled them with the de novo assembler SPAdes with *k*-mer = (15, 21, 25, 31, 35, 39, 45). First, we filtered scaffolds assembled by unmapped reads using BLASTn against BEST195 genes, and we removed scaffolds that had high similarity. Then, a BLASTn search against the nr database was performed for the remaining scaffolds that only targeted *Bacillus subtilis* group (taxid: 653685) with more than 80% identity. For identification of plasmids, we checked the results of BLAST search in terms of sequence similarity and alignment length.

### Data deposition of nucleotide sequence

All whole-genome shotgun sequence reads have been deposited in the Read Archive at DNA Data Bank of Japan (DDBJ) with accession number DRA003017 under DDBJ BioProject PRJDB3484.

## Results

### Genome sequencing

Next-generation sequencing using the Illumina GAII platform was carried out for the eight *B. subtilis* strains, KorC1, MyaA2, LaoA1, ThaB, NepD5, NAFM5, NARUSE, and TAKAHASHI and an average of 54 million reads were obtained. We used only reads in which any base has more than Q30, which means that a base is inferred using base call accuracies of more than 99.9%, and we filtered out the others from datasets. As a result of filtering, an average of 13.6 million reads (about 25% of the original sequencing output) were left, and the average approximate sequence coverage for the whole genome was 190 and the minimum approximate sequence coverage was 68.06 for the NAFM5 strain. Although filtering with Q30 resulted in a pronounced decrease in the number of reads, we thought that these figures were enough for comparative genome analysis based on previous reports [41, 42]. The details of statistics of sequencing data for each strain are shown in Table A in S1 File.

Moreover, we corrected reads employing the Trowel [19] error-correction algorithm. The total number of reads does not change after error correction by Trowel, but the distribution of read length changes because Trowel trims bases off the read ends with the minimum quality value in a given dataset to achieve high-quality results. The statistics of the corrected reads are also shown in Table A in S1 File.

### Reference-assisted assembly and annotation, and comparison with the reference genome

The statistics of the reference-assisted assembly of the eight strains are shown in Table 2. The draft genomes of the eight strains have between 50 and 222 contigs. Based on N50, the maximum contig size, and the number of contigs, strain LaoA1, with the highest sequence coverage, had a good-quality assembly result. In contrast, the assembled genome of strain MyaA2 had a small N50 and maximum contig size in spite of the high sequence coverage. Although strain NAFM5 had the lowest sequence coverage, the assembly result was comparable to that of others. This demonstrates the effectiveness of the reference-assisted assembly because strain NAFM5 is a plasmid-less derivative of the Miyagino (=BEST195) that was used as the reference BEST195 genome. For all eight strains, the total nucleotide length and GC contents of the assembled genomes were close to those of BEST195, which are 4.1Mb and 43.5%, respectively.

**Table 2:** Statics of the reference-assisted assembly and the predicted genes of the eight *B. subtilis* strains.

Statistic	KorC1	LaoA1	MyaA2	ThaB	NepD5	NAFM5	NARUSE	TAKAHASHI
N50	108,025	857,804	69,655	72,647	152,266	114,073	82,070	39,436
total length (bp)	4,077,248	4,202,373	4,036,238	4,111,804	4,035,069	4,007,525	4,079,454	4,045,209
GC content (%)	43.20	43.14	43.28	43.21	43.33	43.42	43.32	43.09
No. contigs	105	50	148	130	83	95	111	222
Predicted CDS	4592	4668	4457	4620	4450	4559	4647	5121
Predicted tRNA	66	72	77	60	67	52	50	26
Predicted rRNA	4	6	6	6	4	3	3	3

**Fig 1:**
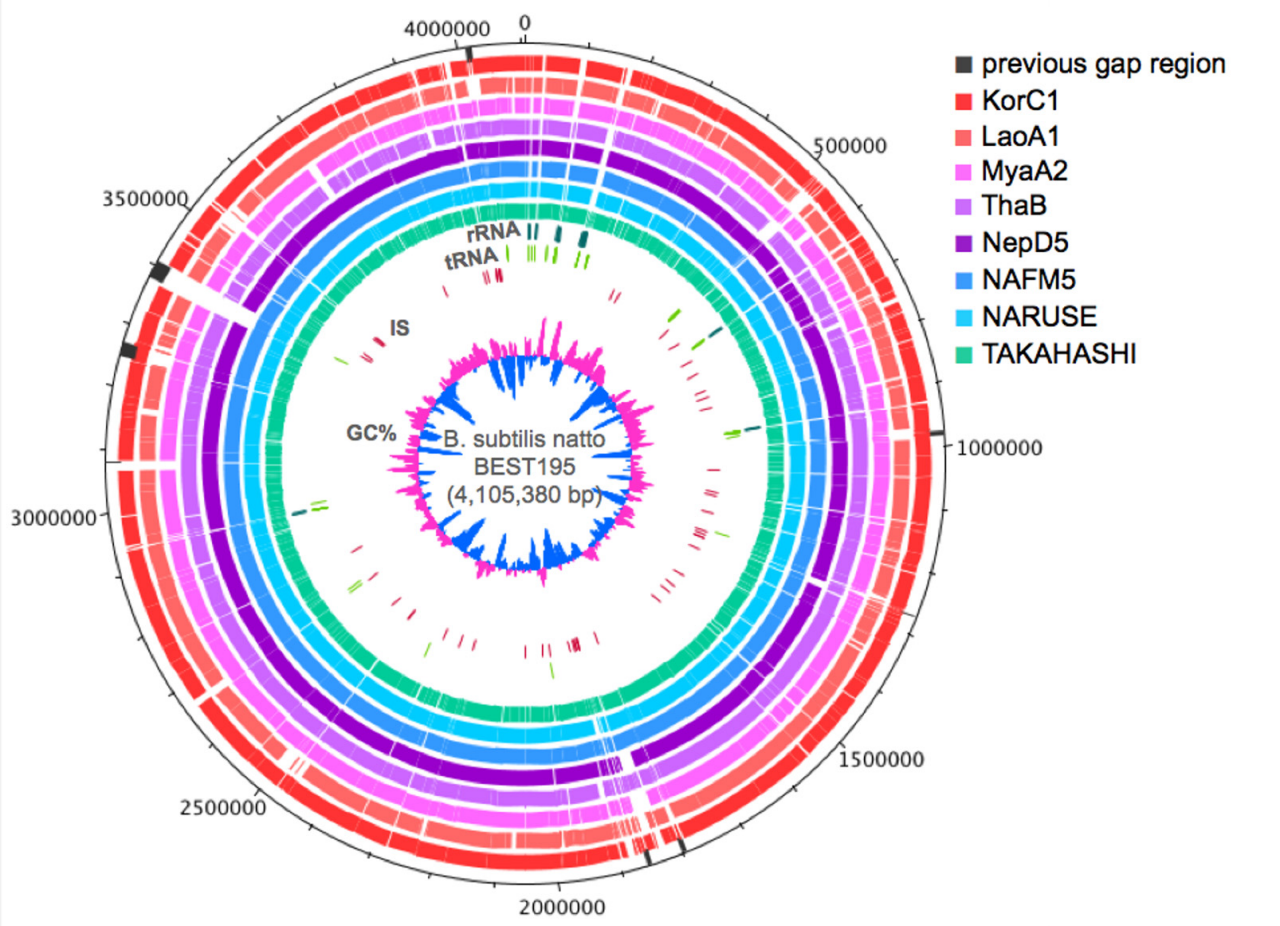
Pairwise sequence alignment between *Bacillus subtilis* BEST195 and the eight *B. subtilis* strains. Each draft genome was aligned to BEST195 using MUMmer. The outside black lines correspond to incomplete regions in the previous BEST195 genome [43], the colored regions in the second to ninth rings from the outside show that scaffolds in the draft genome were aligned to the BEST195 genome. The inner circle at the center displays the G+C content (window size = 10,000bp; step size = 200). This genetic map was generated using DNAPlotter.

Fig 1 shows aligned regions of the BEST195 genome with each draft genome using MUMmer. In our previous study about whole-genome resequencing of BEST195 [5], we discussed gap (incomplete) regions in the first draft genome sequence that previously had been sequenced using very short reads generated from Illumina GAII [43], and we showed that these regions were attributed to GC bias and repetitive sequences. It is known that these regions are difficult to assemble with only short reads. The previous gap regions are indicated as black lines in Fig 1. Many of the unaligned regions (white regions in Fig 1) correspond to the previous gap regions, and regions including transposases of ISs and rRNA genes, which are also difficult to assemble because of the characteristics of repetitive sequences and patterns. When a sequence differs from the reference sequence or *de novo* assembly in the first step does not work well, the regions cannot be covered even though we used the reference-assisted process. Therefore, there is a high possibility that unaligned regions are regions of repetitive sequences that could not be assembled with short reads or deleted regions in the strains.

The numbers of predicted CDS, tRNA, and rRNA genes for the draft genomes are also shown in Table 2. Between 4450 and 5121 protein genes were predicted for the eight strains using Glimmer. One copy of the rRNA cluster (5s, 23s, and 16s rRNA), and 52, 50, and 26 tRNA genes were predicted for three Japanese strains, NAFM5, NARUSE, and TAKAHASHI, respectively. For strains KorC1 and NepD5, 2 copies of the 5s rRNA gene, 1 copy of the 23s and 16s rRNA genes, and 66 and 67 tRNA genes were predicted, respectively. For the other strains, LaoA1, MyaA2, and ThaB, 4 copies of the 5s rRNA gene, 1 copy of the 23s and 16s rRNA genes, and 72, 77, and 60 tRNA genes were predicted, respectively. Compared with the numbers of the rRNA cluster and tRNA genes in the BEST195 genome, which are 10 copies (total 30 rRNA genes) and 87, those of numbers in the draft genomes were small. This is attributed to the fact that tRNA and rRNA genes of BEST195 are included in unaligned regions in Fig 1, and these regions could not be assembled from short reads.

Using the RBH method with BLASTx, we investigated orthologous genes to BEST195 and 168. The non-Japanese strains KorC1, LaoA1, MyaA2, ThaB, and NepD5 have orthologs to 83.07, 77.10, 82.22, 82.49, and 87.14% of the BEST195 genes, respectively. The Japanese strains NAFM5, NARUSE, and TAKAHASHI have orthologs to 90.18, 87.55, and 73.64% of the BEST195 genes, respectively. The reason why strain TAKAHSHI had the smallest number of orthologous genes to BEST195 is that strain TAKAHASHI had a lot of small scaffolds in the assembled draft genome, resulting in many predicted genes with a small length. When we considered only the alignment length for the draft genome genes, strain TAKAHASHI had almost the same number of orthologous genes to BEST195 as the other Japanese strains (94.12, 94.14, and 93.94% for NAFM5, NARUSE, and TAKAHASHI strain, respectively). We performed the same procedure against *B. subtilis* 168, and we found that only strain LaoA1 had more orthologous genes to 168 than BEST195, which was 85.76% of the 168 genes. This suggests that strain LaoA1 is related more closely to 168 than BEST195.

### Insertion sequence

We investigated the transposases of five types of ISs, that is, IS*4Bsu1*, IS*Bma2*, IS*643*, IS*256Bsu1*, and IS*Lmo1*, and a putative transposase. Performing the BLASTx search with the predicted genes in the draft genomes against BEST195, many genes had similarity with transposases of the ISs. The number of IS and details are shown in Table B in S1 File. As we mentioned in the above section, because the regions including transposase are difficult to be assembled with short reads, we did not consider the alignment length. Therefore, although BLASTx hits with a short alignment length were included, strain ThaB had the highest number of genes similar to transposases of BEST195, and strain NepD5 also had many genes with similarity to the transposases. It was experimentally reported in [11] that the IS frequency was high in strains that were used for fermented soybeans in Thailand and Nepal.

The scaffolds in the draft genomes containing genes similar to transposases were aligned to the gnome sequence of BEST195. Then, scaffolds that could not be aligned to the BEST195 genome could be regarded as nonexistent transposases in BEST195.

**Fig 2:**
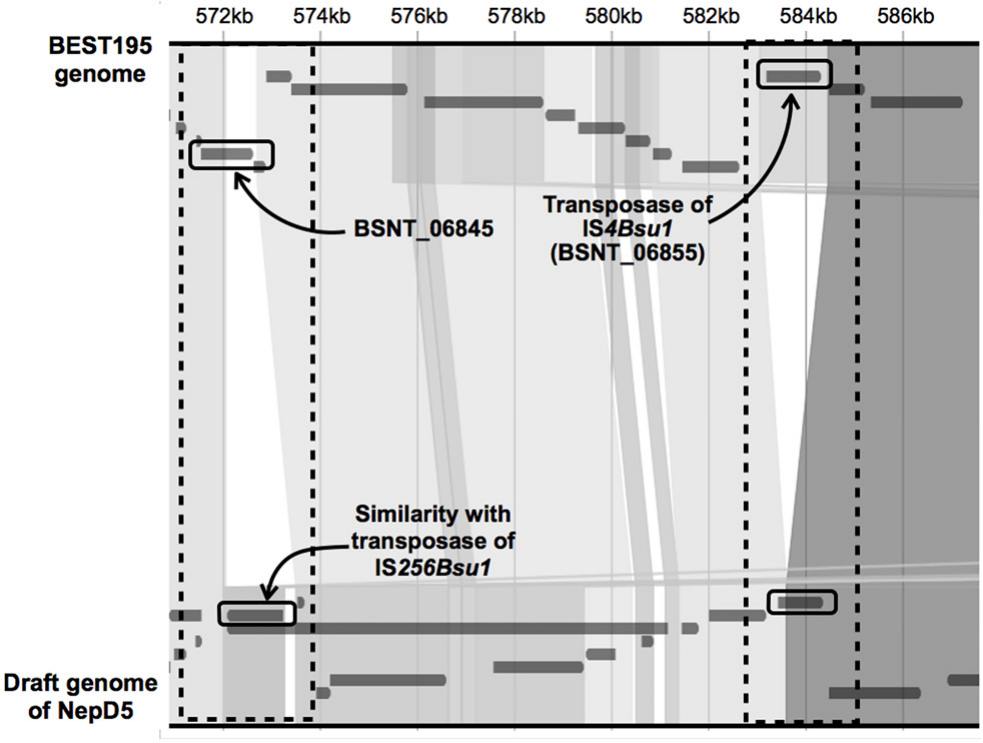
Insertion of transposase into the draft genome of strain NepD5 and the *B. subtilis* BEST195 genome. The NepD5 draft genome is aligned to BEST195 using Murasaki. The similar depth connections of both genomes indicate that they have conserved sequences, and the white regions indicate non-conserved sequences. In the NepD5 draft genome, the sequence similar to the transposase of IS*256Bsu1* is inserted into a gene (BSNT_06845) of BEST195 (left black dashed line), and in the BEST195 genome, the transposase of IS*4Bsu1* is inserted into a coding region of the NepD5 genome (right black dashed line).

Here, we provide one example of these transposases in Fig 2. The coding region in strain NepD5 with length of 1155 bp had similarity to a transposase of IS*256Bsu1* with length of 1369 bp, and it was aligned to a position in BSNT_06845 that encodes a hypothetical protein. Although the function of BSNT_06845 has not been identified yet, *yddC* and *yddD*, which are located upstream and downstream of BSNT_06845, are annotated as transcriptional regulator and mobile element region, respectively. In Fig 2, we can also see another example of the insertion of a transposase in the NepD5 genome. BSNT_06855 is a transposase of IS*4Bsu1*, and it was aligned to a coding region of strain NepD5. This coding region with length 927 bp has similarity to a gene encoding a hypothetical protein of *B. subtilis*. The examples shown in Fig 2 exhibit frequent occurrence of transposition of IS in the soybean-fermenting *B. subtilis* genome, and there is a possibility that the above insertion of transposases is related to a difference of phenotypic trait between strain NepD5 and BEST195.

### Mapping and variant calling

Filtered-corrected short reads sequenced from the eight *B. subtilis* strains were mapped to the BEST195 genome sequence using BWA. An average of 86.2% of reads were mapped to the BEST195 genome with an average of 193.6-fold coverage across the entire genome. Based on the mapping result, SNPs and INDELs were detected for all strains using GATK, and effect impacts of each variant on a genome were estimated by SnpEff. The statistics of the mapping and variant calls for each strain are summarized in Table C in S1 File.

**Fig 3:**
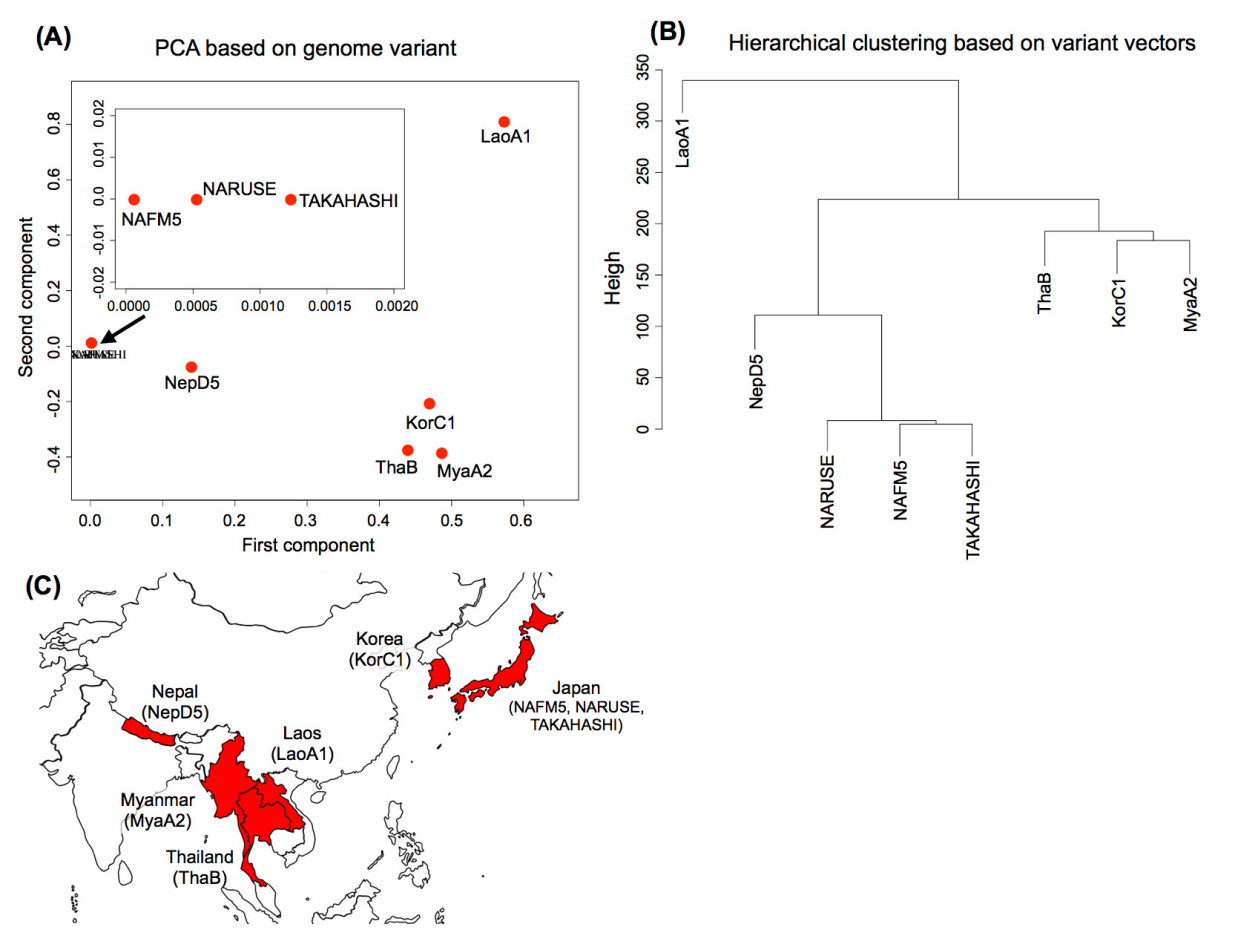
The results of PCA and hierarchical clustering based on variant vectors. (A) Biplot of principal component analysis based on variant vectors. The dots show the eight *B. subtilis* strains, and the upper left image is an enlarged image focused on the three Japanese strains located near (0, 0). The fist principal component features the non-Japanese strains, and the second principal component can be regarded as a feature to distinguish strain LaoA1 and the other non-Japanese strains. (B) Hierarchical clustering of the eight *B. subtilis* strains based on the Euclidean distance between variant scores of each strain using the furthest neighbor method. The different cluster indicates that strains have different variant score patterns. (C) Geographical location of each country.

To score and vectorize detected variants (see Material and Methods), we performed PCA and hierarchical clustering analysis, and the results are shown in Fig 3. The principal components obtained in PCA were transformations of variant score vectors by a linear combination that was chosen to maximize the variance of the score vectors of all eight strains. As shown in Fig 3-A, the first principal component (contributing rate: 51%) indicates a feature of the non-Japanese strains, and the second principal component (contributing rate: 19%) can be regarded as a feature to distinguish strain LaoA1 from the other non-Japanese strains. The Japanese strains converged and formed a small cluster. For the first principal component, principal scores are high for genes BSNT_09336, BSNT_09102, and BSNT_09338, which mean that these genes contribute to the first principal component. Although they all are annotated as genes encoding hypothetical proteins and their function is not identified yet, BSNT_09337 and BSNT_09100, which are located near these genes, are annotated with GO terms related to membrane and DNA binding, respectively. Genes BSNT_08913, BSNT_06665, and BSNT_08139 highly contributed to the second principal component. The functions of these genes are also unclear, but BSNT_08139 is annotated with a GO term of motor activity. BSNT_06664, which is located upstream of BSNT_06665, is annotated with a GO term of transferase activity, *comGA* (BSNT_08912) encodes a competence protein GA, and BSNT_08914 is annotated with a GO term related to adenyl nucleotide binding.

We also performed a hierarchical clustering based on variant vectors to investigate differences in gene variants. As the clustering result in Fig 3 shows, the Japanese strains were grouped into a single cluster as in PCA, and strain NepD5 was in another cluster close to the Japanese strains. Strains ThaB, KorC1, and MayA2 fell into a cluster, and strain LaoA1 was in a cluster independent of any other strains. It is obvious from Fig 3-C that genetic differences of the non-Japanese strains are not associated with their geographical locations.

Next, to investigate functional differences across the eight strains, we focused on genes with variants predicted to have high- and moderate-impact effects that have effects on protein functions. A total of 2233, 1416, 1147, and 1214 genes were listed for strains LaoA1, KorC1, MyaA2, and ThaB, respectively, and 365 genes were listed for strain NepD5. On the other hand, for the Japanese strains, 6 and 23 genes were identified as genes with influential variants in strains NARUSE and TAKAHASHI, respectively, and no genes with high and moderate variants were found in strain NAFM5. These listed genes were investigated in terms of GO annotations along with the reference genome. For GO terms associated with “biological process”, the number of genes with GO terms of “metabolic process”, “cellular process”, and “single-organism process” were high for both Japanese and non-Japanese strains. Details of the GO term analysis are shown in Table D in S1 File. Although many analyses can be done with the detected genes with variants, to discuss in depth about the most interesting aspect of *B. subtilis*, in the section below, we investigated the detected variations in terms of biotin synthesis, the subtilisin NAT, the production of *γ*-PGA, and the motility of *B. subtilis*.

#### Biotin auxortophy of *B. subtilis* natto

**Fig 4:**
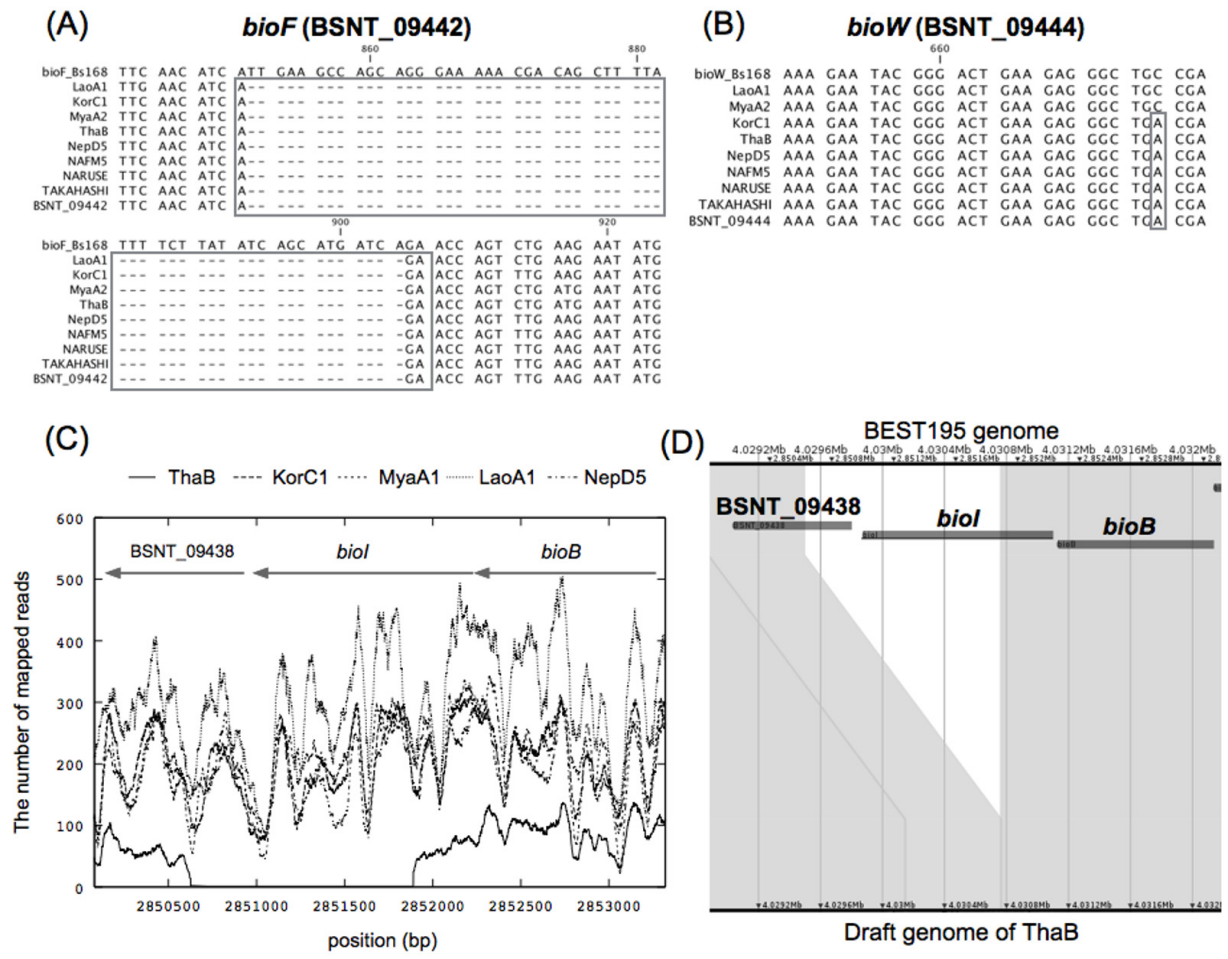
Variant analysis of the *bio* operon. The genome sequences of eight *B. subtilis* strains were obtained via the variant data, and the multiple sequence alignments of them with BEST195 and *B. subtilis* 168 were performed focusing on BSNT_09442 (A) and BSNT_09444 (B). A large nucleotide deletion in *bioF* and a nonsense mutation in *bioW* are found in BEST195. All eight *B. subtilis* strains have the same deletions in *bioF* as BEST195, while the nonsense mutation in *bioW* was not found in strains LaoA1 and MyaA2. (C) The number of mapped reads of the non-Japanese strains against BSNT_09438, *bioI* and *bioB* of BEST195. For only strain ThaB, no read was mapped to the end of BSNT_09438 and most of *bioI*. (D) The pairwise sequence alignment of the draft genome of strain ThaB and BEST195. An unmapped region in (C) was deleted in the genome sequence of strain ThaB.

Biotin is synthesized via the *bio* operon (*bioWAFDBI*) in *B. subtilis*. In the natto-fermenting strains, the *bioB* gene is functionally expressed, but the *bioW* gene and *bioF* gene are defective because of a nonsense mutation and a large deletion, respectively [44]. These defects result in biotin auxotrophy of the natto-fermenting strains. According to the experimental studies, the biotin depletion condition leads to an overproduction of L-glutamic acid that is a component of *γ*PGA [45, 46]. Therefore, biotin auxotrophy might be related to transcriptional regulation that is favorable for *γ*PGA synthesis and natto fermentation [10]. The multiple sequence alignment of *bioF* and *bioW* in the genomes of the eight strains, BEST195, and 168 showed that all eight strains had the same deletion in *bioF* as BEST195. However, strains LaoA1 and MyaA2 did not have the nonsense mutation in *bioW* that was also missing in strain 168 (Fig 4). ThaB had the nonsense mutation in *bioW*, but this strain is biotin prototrophic [11]. Thus, it is assumed that biotin auxotrophy of the natto-fermenting strains depends mainly on the deletion in *bioF*. The non-Japanese strains excluding NepD5 have many nucleotide changes in the *bio* operon. Especially, MyaA2 has a variant causing frame shift in *bioD* that encodes a dethiobiotin synthetase. The positions and details of these variants are shown in Table E in S1 File. Additionally, in the ThaB strain, no read was mapped to *bioI* and a portion of BSNT_09438 (Fig 4). *bioI* encodes a biotin biosynthesis cytochrome P450 protein, and BSNT_09438 encodes a hypothetical protein and corresponds to *ytbQ* in strain 168. Pair-wise alignment of BEST195 and the draft genome of strain ThaB using Murasaki also showed the deletion of these regions (Fig 4). Thus, we inferred that biotin synthetic organization of strains LaoA1, MyaA2, and ThaB are different from those of other strains, and these changes might result in different biotin metabolism and growth conditions.

#### Production of subtilisin NAT

Subtilisin NAT (formerly designated as nattokinase) is an extracellular enzyme secreted by *B. subtilis* (natto) [47], and belongs to the alkaline serine protease family. Subtilisin NAT is considered to be the most important enzyme for the characteristic taste and flavor of natto, and a gene encoding subtilisin NAT was determined to be *aprN* [48]. Focusing on *aprN* and neighboring genes, three nucleotide changes were found to be common in *aprN* in strains LaoA1, KorC1, and MyaA2. However, a thymine-to-cytosine nucleotide change in *aprN* that leads to a non-synonymous mutation (asparagine to serine) was found only in strain ThaB. Some nucleotide changes were also found in genes located upstream and downstream of *aprN* in strains KorC1, LaoA1, MyaA2, ThaB, and NepD5. The details of these variations are shown in Figure A and Table F in S1 File. It has been reported that some *B. subtilis* strains isolated from “Tua nao” exhibited higher value of production of subtilisin NAT and PGA than the Japanese commercial strain used in natto production [49]. Therefore, there is a possibility that the nucleotide changes uniquely found in strain ThaB are related to the high productivity of subtilisin NAT, and the experimental verifications of these variants may provide an insight into the difference on the subtilisin NAT production.

#### Production of *γ*PGA

The production of *γ*PGA has been extensively studied because *γ*PGA plays an important role in industrial production and medical treatment. In strain 168, which is incapable of producing *γ*PGA, a single nucleotide is substituted from cytosine to thymine in the promoter region of *degQ* and a single adenine is inserted into the coding region of *swrAA*. These two nucleotide substitutions are specifically present in the BEST195 genome, and *swrAA* has been reannotated as *yvzD*.

First, we confirmed whether or not the eight strains have the same substitutions as BEST195. No different substitution was found in *degQ*; that is, all eight strains have the same substitution in *degQ*. However, two nucleotides deletion was found in *swrAA* only of LaoA1, while the other strains have the same sequence as BEST195. This two nucleotides deletion also causes the pseudogenization of *swrAA* in common with BEST195 and other strains. The multiple sequence alignment of *swrAA* (*yvzD*) from the eight strains, BEST195, and 168 is shown in Figure B in S1 File.

Cell density-dependent phenotypes of *B. subtilis* are regulated by the ComQXPA quorum-sensing system, which involves the ComP-ComA two-component signal transduction system [50, 51]. It is known that the synthesis of *γ*PGA in *B. subtilis* (natto) is also controlled by this system [52]. *B. subtilis* (natto) uses *γ*PGA as an extracellular nutrient reservoir. A high cell density, which is a sign of overhanging starvation, triggers the synthesis of *γ*PGA [53, 54]. ComQXPA quorum-sensing machinery of *B. subtilis* is known to have divergent structure as for the extracellular signaling peptide ComX and the N-terminal ComX binding domain of the membrane receptor kinase ComP [43, 52].

All strains have the same variations as BEST195 in the DNA region of ComQXPA, and three nucleotide changes that lead to non-synonymous mutations were found in strains KorC1, LaoA1, and MyaA2 at the end of the coding region of *comP* (Figure C and Table G in S1 File). One of these nucleotide changes found in KorC1, LaoA1, and MyaA2 corresponded to the same variant in 168 strain. Of the other two nucleotide changes one was unique for strain KorC1 and one was commonly detected in KorC1 and MyaA2. The N-terminal part of the coding region of *comP* is a transmenbrane sensor domain that binds *comX*, while the end part is a kinase domain that is localized in the cytoplasm [52]. Therefore, these nucleotide changes have a potential impact on the phosphorylation of *comA*. Details of variations and the multiple sequence alignment of sequences corresponding to the nucleotide changes in *comP* are shown in Figure C and Table G in S1 File, respectively. The cell density-dependent phenotype and the motility of bacteria are related to each other. In the following section, we focused on the relationship between the motility of *B. subtilis* and the *γ*PGA production.

#### Motility of *B. subtilis*

**Fig 5:**
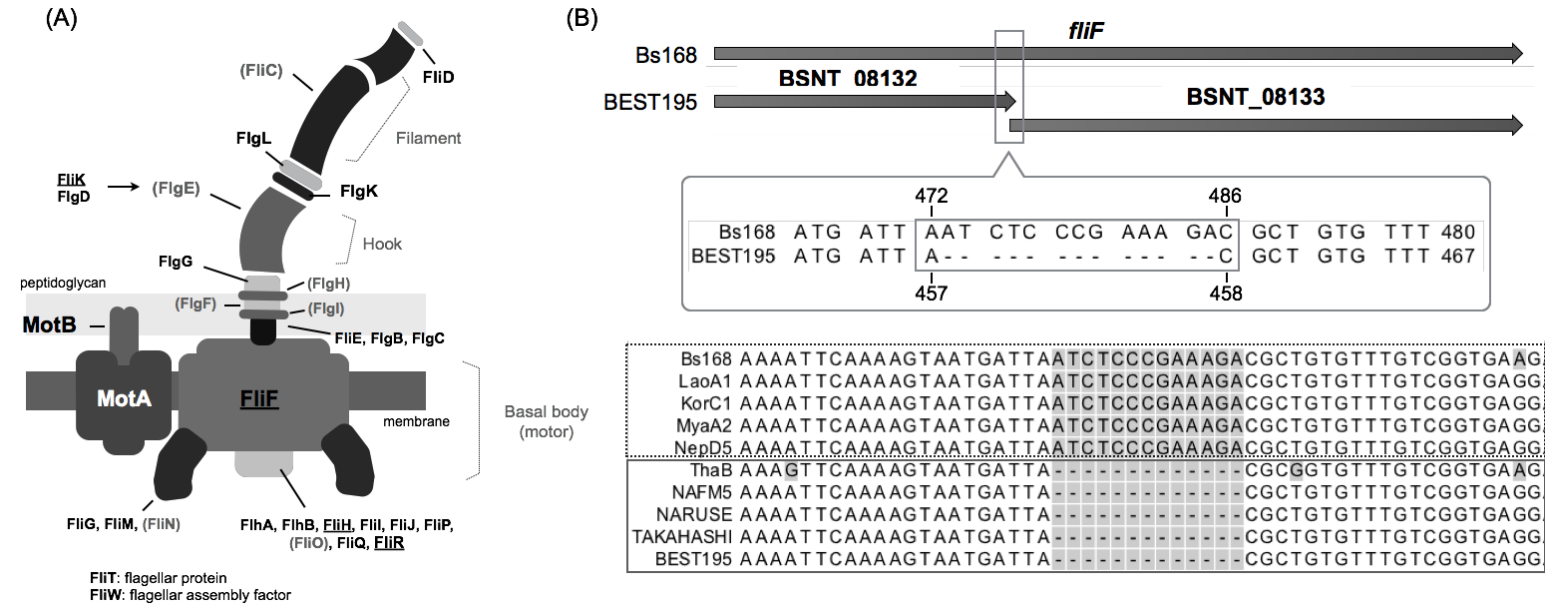
Variant analysis focused on flagellum. (A) Diagram of the bacterial flagellum. Underlined proteins are encoded by a gene that was reannotated in the BEST195 genome. Proteins written in gray and in brackets are not annotated in BEST195 and *B. subtilis* 168; these proteins were removed from the analysis. (B) The nucleotide deletions in *fliF* that encodes FliF and the sequence alignment of *fliF* and corresponding genes BSNT_08132 and BSNT_01833. The same deletion in *fliF* that is in BEST195 was found in strains ThaB, NAFM5, NARUSE and TAKAHASHI, while it was not found in other non-producing *γ*PGA strains.

Recent studies reported a relationship between the motility and *γ*PGA production of *B. subtilis* [55, 56]. These studies suggested that flagellar rotation negatively affects *γ*PGA synthesis, and a lack of motility might enhance *γ*PGA synthesis. The bacterial flagellum is a complex molecular machine composed of about 30 different proteins. It is organized into three main parts: basal body (motor), hook, and filament [57]. A diagram of the bacterial flagellum (for *B. subtilis*) with 31 proteins is shown in Fig 5-A. We investigated genes that encode 24 proteins given in Fig 5, excluding proteins that encode genes that are unannotated in BEST195 and 168.

First, we focused on *motA* and *motB*, which encode the MotA and MotB flagellar stator proteins in *B. subtilis*. No nucleotide change was found in *motB* in the eight strains, but two nucleotide changes were found in *motA* in strain LaoA1. These changes cause a codon insertion (glutamate) and a non-synonymous mutation (alanine to threonine); strain 168 has the same sequence as strain LaoA1 (Table H in S1 File). Among the 24 genes given in Fig 5-A, some nucleotide changes were found in 11 genes only in LaoA1 including the above *motA*, and no change was found in 8 genes of the eight strains. Some common changes in more than one strain were also found: an adenine-to-thymine nucleotide change in *flgK* encoding FlgK was identified in strains KorC1 and LaoA1, a thymine-to-adenine nucleotide change in *flgC* encoding FlgC was identified in strains LaoA1 and ThaB, and a thymine-to-adenine nucleotide change in *flhA* encoding FlhA was identified in strains LaoA1, KorC1, MyaA1, and ThaB. We also observed a cytosine-to-guanine nucleotide change in *flhA* and a guanine-to-adenine nucleotide change in *flgB* encoding FlgB in strain ThaB. The details of variation in 24 genes are shown in Table H in S1 File.

Focusing on *fliF*, which encodes the flagellar MS-ring protein FliF. A 13-bp fragment encoding 5 amino acids in *fliF* was largely deleted, and *fliF* is separated into two genes, BSNT_08132 and BSNT_08133 in the BEST195 genome. This deletion is estimated to deactivate *fliF* in BEST195. It should be noted that the same deletions were found in strain ThaB and the three Japanese strains, but the other strains had the same sequence as strain 168 (Fig 5-B). According to previous experimental data [11], strains LaoA3, which was isolated from the same source as LaoA1, MyaA2, and NepD5 did not produce *γ*PGA, but strain ThaB did. In other words, a common difference in the non-Japanese strains excluding strain ThaB might have an influence on the production of *γ*PGA. Additionally, in the experiment in [56], *γ*PGA overproduction was observed in mutants defective in flagellar basal body assembly, including a mutation in *fliF*. Although the production of *γ*PGA by KorC1 was not indicated in [11], it is highly likely that the 13-bp deletion of *fliF* causes the strain to be nonmotile and improves *γ*PGA yields. Moreover, this result strongly supports the suggestions from the experimental studies at the nucleotide sequence level. The common deletion in *fliF* among *B. subtilis* strains producing *γ*PGA is a previously unreported variation.

Additionally, as described previously, the production of *γ*PGA in *B. subtilis* isolated from “Tua nao” (the same source foods as ThaB) were higher than that of the Japanese natto-fermenting strains. Thus, it is possible that the nucleotide changes in *flhA* and *flgB* that are uniquely found in strain ThaB trigger the overproduction of *γ*PGA.

### MLST analysis

There are many methods to investigate phylogenetic relationship among bacteria strains. Since whole genome of eight strains were sequenced in this study, it is possible to employ phylogenetic analysis with whole-genome, such as the assembled draft genome sequences and the sequences constructed from detected variants. However, as shown in Figs 1 and 4, incomplete regions were remained on these genomes. Therefore, we employed MLST analysis that uses only seven housekeeping genes selected with consideration for evolutionary rate as phylogenetic analysis in this study.

**Fig 6:**
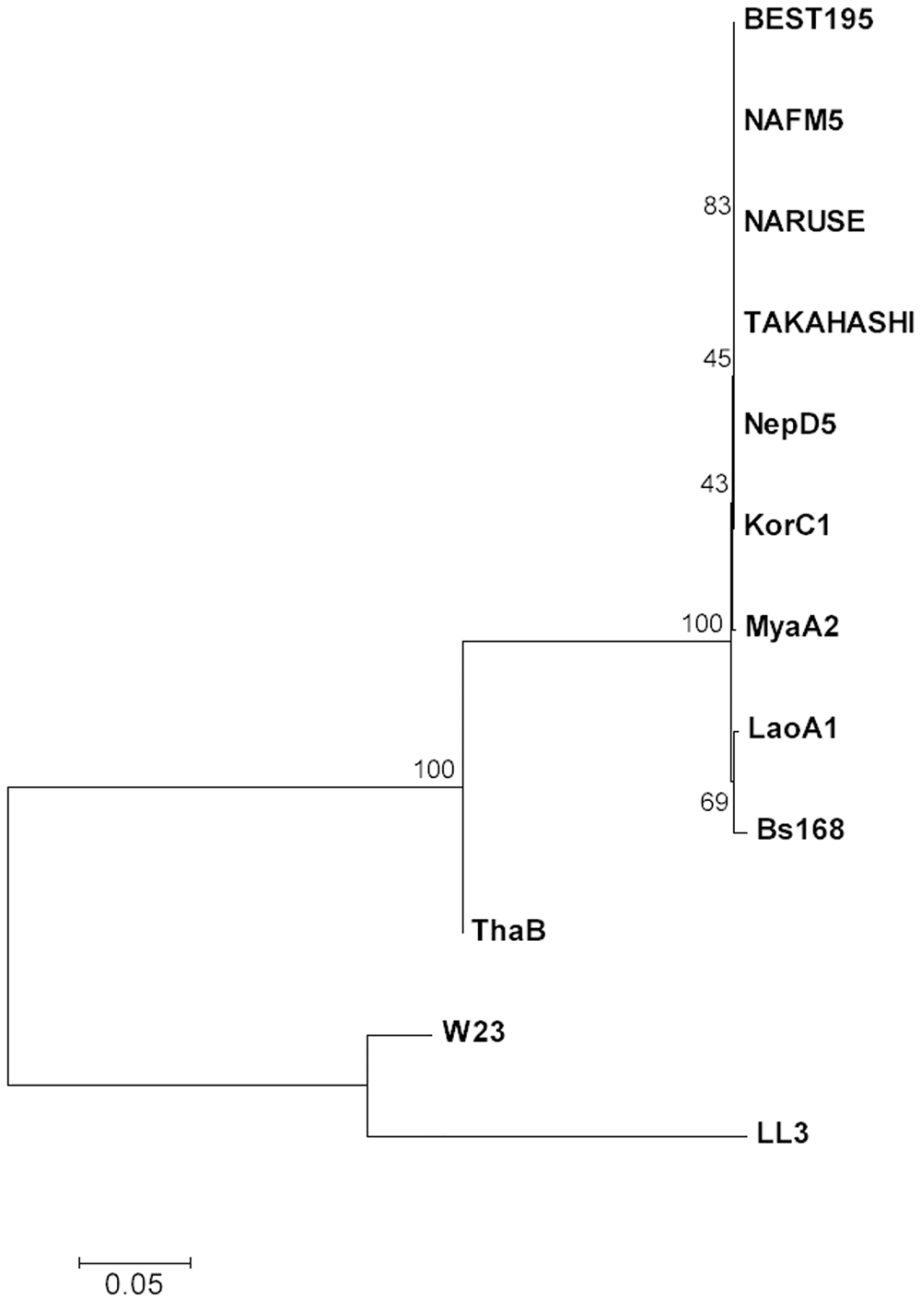
Phylogenetic relationship between the eight *B. subtilis* strains isolated from non-salted soybean foods, *B. subtilis* BEST195, 168, W23, and *B. amyloliquefaciens* LL3. An unrooted phylogenetic tree was generated using the neighbor-joining method based on the seven genes of multilocus sequence typing analysis. The numbers along branches indicate bootstrap percentages.

The housekeeping genes *glpF*, *ilvD*, *pta*, *purH*, *pycA*, *rpoD*, and *tpiA* were chosen for MLST analysis for *B. subtilis* [38]. The internal fragment sequences of these genes were identified using the database and concatenated into a sequence for each strain. Using MEGA 6.0, we analyzed the phylogeny of the eight strains, BEST195, 168, W23, and *B. amyloliquefaciens* LL3. Fig 6 shows an unrooted phylogenetic tree of the eight *B. subtilis* strains of this study, BEST195, *B. subtilis* 168, W23, and *B. amyloliquefaciens* LL3. BEST195, NepD5, and three Japanese strains (NAFM5, NARUSE and TAKAHASHI) formed a tight cluster. These strains are more evolutionary close to each other than the other non-Japanese strains, and these five strains have the same allelic profile for all seven genes and sequence type by MLST analysis. (The allelic profiles for all strains are shown in Table I in S1 File.) The same sequence type was also shown in 12 *B. subtilis* strains registered in PubMLST of *B. subtilis*, and most of the annotated strains were isolated from soybeans in Japan. The strain LaoA1 formed a cluster with 168. This is a reasonable result because they both are incapable of producing *γ*PGA and have homology to each other as shown in the ortholog analyses. The ThaB strain was thought to be apart from other *B. subtilis* strains earlier than other strains, and it is inferred that a strain with a survival advantage in terms of biotin synthesis and production of *γ*PGA was survived in Thailand. *B. amyloliquefaciens* LL3 was isolated from a fermented food (Korean bibimpa) and synthesizes *γ*PGA [18], but it was on the same branch as W23. From these results, the evolutionary process of the soybean-fermenting strains was thought to be independent from the ability of fermentation and the production of *γ*PGA.

### *de novo* Assembly with unmapped reads

To investigate extrachromosomal DNAs such as a plasmid and the differences from the BEST195 genome, we assembled unmapped reads, which were not used in the variant analysis, into longer scaffolds using SPAdes. A small number of scaffolds with more than 1kb of sequence from the Japanese strains and between 24 and 935 scaffolds for the non-Japanese strains were obtained. Using BLASTn against the BEST195 genes, all scaffolds of strain NAFM5 and most of the scaffolds of the other seven strains were identified as sequences in the BEST195 genome. Between 4 and 15 scaffolds of the seven strains remained without similarity to the BEST195 genes; they were used in a BLASTn search of the nr database that only targeted the *Bacillus subtilis* group. The statistics of *de novo* assembly and BLASTn search are shown in Table 3.

**Table 3:** The results of *de novo* assembly and BLASTn search against the BEST195 genes.

	KorC1	LaoA1	MyaA2	ThaB	NepD5	NAFM5	NARUSE	TAKAHASHI
No. unmapped reads	1,934,400	5,654,426	1,787,502	3,370,384	742,132	77,136	686,796	469,936
No. SCF (≥ 1kbp)	76	132	67	935	24	5	11	8
No. SCF w/o similarity to BEST195	4	10	15	11	5	0	7	5

SCF: Assembled genomic scaffold

All remaining scaffolds of the NARUSE strain were identified as a plasmid with the same sequence as *B. subtilis* (natto) plasmid pL20 (65,774 bp; GenBank accession number AB615352), and the scaffolds of TAKAHASHI were identified as plasmids with the same sequence as pLS20 and B. subtilis natto plasmid pBEST195S (5838 bp; GenBank accession number AP011542). BEST195 contains two plasmids, pBEST195L and pBEST195S; pBEST195L is similar to pLS20 [58]. Therefore, it is thought that stain TAKAHASHI contains the same plasmids as BEST195 and that strain NARUSE contains a plasmid with the same sequence as pLS20 (pBEST195L).

Most of the remaining scaffolds of the non-Japanese strains were also identified as plasmids. Although the other scaffolds, which were not identified as plasmids, had similarities with some sequences of other *B. subtilis* and *B. amyloliquefaciens* at the nucleotide level, many of them have only partial match with fractions of scaffolds and some of them had similarities with transposases, which have repetitive sequences. Thus, we could not eliminate the possibility of misassembly and did not discuss them into detail. The BLASTn results suggested that stain KorC1 contains a plasmid with the same sequence as pBEST195S and a plasmid similar to *B. subtilis* plasmid p1414 (7949 bp; GenBank accession number AF091592), stain LaoA1 contains a plasmid with the same sequence as p1414 and a plasmid similar to *B. amyloliquefaciens* LL3 plasmid pMC1(6758 bp; GenBank accession number CP002635), and strain MyaA2 contains plasmids similar to pBEST195S, p1414, and *B. subtilis* ATCC 15841 plasmid pPL1 (6704 bp; GenBank accession number DQ140187). Strain ThaB seems to contain plasmids with the same sequences as pMC1 and p1414, and strain NepD5 is thought to contain a plasmid with the same sequence as pBEST195S. The identified plasmids for each strain are summrized in Table 4.

**Table 4:** The plasmids of BEST195 and the identified plasmids in the eight *B. subtilis* strains.

	BEST195	KorC1	LaoA1	MyaA2	ThaB	NepD5	NAFM5	NARUSE	TAKAHASHI
pLS20 [pBEST105L]	✓							✓	✓
pBEST195S	✓	✓		✓		✓			✓
*B. subtilis* p1414		✓	✓	✓	✓				
*B. amyloliquefaciens* pMC1			✓		✓				
*B. subtilis* pPL1				✓					

## Conclusion

This study performed whole-genome shotgun sequencing of eight *B. subtilis* strains isolated from non-salted fermented soybean foods in Southeast Asia, and it investigated genetic differences among them using comparative genomics approaches. Using comparative variant analysis, we showed the differences in biotin auxotrophism for the strains, and examined potential nucleotide changes that improve the production of subtilisin NAT (nattokinase) and *γ*PGA. Furthermore, our results suggested that the deletion in *fliF* encoding FliF, which constitutes the flagellar basal body, is related to the production of *γ*PGA. We hope that the genomic differences detected in this work promise new insights into phenotypic characteristic of *B. subtilis*.

Although the natto-fermenting strains are classified as *B. subtilis* species in the National Center for Biotechnology Information Taxonomy, there is no sharp taxonomic distinction between the natto-fermenting *B. subtilis* strains and other *B. subtilis* strains. Moreover, it is known that the production of *γ*PGA alone is not a predictor of the ability to ferment natto because many *γ*PGA-positive strains cannot be used for natto production.

Phylogenetic analysis revealed that the natto-fermenting Japanese strains fell into a tight cluster in the phylogenetic tree as previously described [10]. However, when strains that were isolated outside Japan were included in the analysis, the strains with the ability to ferment soybeans did not fall into a single cluster in the phylogenetic tree. Thus, this study showed that *B. subtilis* strains that could be used in fermented soybean production could not be classified into a single taxonomic group based on lineage analysis.

There are some discussions about the origin of non-salted fermented soybean foods [59]. Our results did not show correlations between geographical location and phylogenetic history for *B. subtilis* strains isolated from non-salted fermented soybean foods, and also suggested that strain ThaB, which was isolated from fermented food in Thailand, followed a different diffusion process than other strains. An comparison of genome sequences constructed from short sequence reads revealed that strain LaoA1 was different from the other strains but similar to the standard laboratory strain *B. subtilis* 168. The detailed analysis will be needed to understand this result, but there is a possible involvement of the cultural history of the non-salted fermented soybean foods. The genetic differences revealed in this study provide a clue to understand the origin and routes of non-salted fermented soybean foods in Southeast Asia.

The expected merits of this study are not merely confined to industrial applications. Natto draws attention as a health food and some efforts have been made to develop natto to suit each person’s taste through trial and error; for instance, natto with a decreased aroma or the softening of beans for people who experience difficulty in mastication or deglutition. There are further possibilities for the flexible production of natto using correlation analysis of the genotype and phenotype of fermented soybean foods based on the comparative genome analysis of this study.

## Supporting Information

S1 File Additional information of the results. The details of sequencing output (**Table A**). The results of BLASTn hits with the transposase of the insertion sequence (**Table B**). The details of mapping and variant calls (**Table C**). The details of GO term counts for genes with variations (**Table D**). The details of variations in the *bio* operon (**Table E**). The details of variations in *aprN* and neighboring genes (**Figure A** and **Table F**). The result details for the analysis of the productivity of *γ*PGA (**Figure B**, **Figure C**, and **Table G**). The result details for the analysis of the motility of *B. subtilis* (**Table H**). The details of the MLST analysis (**Table I**).
(PDF)
