# Supplementary material for: Whole-Genome Sequencing and Comparative Genome Analysis of Bacillus subtilis Strains Isolated from Non-Salted Fermented Soybean Foods
Source: PLoS One. 2015 Oct 27;10(10):e0141369. doi: 10.1371/journal.pone.0141369 (PMC4624242; doi:10.1371/journal.pone.0141369)
Supplement: S1 File — The details of sequencing output (Table A). The results of BLASTn hits with the transposase of the insertion sequence (Table B). The details of mapping and variant calls (Table C). The details of GO term counts for genes with variations (Table D). The details of variations in the bio operon (Table E). The details of variations in aprN and neighboring genes (Figure A and Table F). The result details for the analysis of the productivity of γPGA (Figure B, Figure C, and Table G). The result details for the analysis of the motility of B. subtilis (Table H). The details of the MLST analysis (Table I). (PDF) [file pone.0141369.s001.pdf]

Additional information of the results

Whole-genome sequencing and comparative genome analysis of *Bacillus subtilis* strains isolated from non-salted fermented soybean foods

Mayumi Kamada, Sumitaka Hase, Kazushi Fujii, Masato Miyake, Kengo Sato,

Keitarou Kimura, Yasubumi Sakakibara

Contents:

The result details for "Genome sequencing" 2

The result details for the analysis of "Insertion sequence"............................ 2

The details for "Mapping and variant calling" 3

The result details for the analysis "Biotin auxortophy of *B. subtilis* natto" 4

The result details for the analysis "Production of subtilisin NAT" 7

The result details for the analysis "Production of γPGA" 11

The result details for the analysis "Motility of *B. subtilis*".............................13

The result details for MLST analysis..17

The result details for "Genome sequencing"

Table A. Details of sequencing output and error-corrected reads by Trowel

	KorC1	LaoA1	MyaA2	ThaB	NepD5	NAFM5	NARUSE	TAKAHASHI
Original sequencing output								
No. paired-reads	48,140,926	73,283,254	50,775,916	37,352,714	57,369,358	1,471,340	80,341,082	74,090,210
Read length	58	58	58	58	56	56	56	56
Q30 (%)	32.03	34.20	31.54	33.18	27.93	43.49	16.12	8.14
Inferred coverage*	217.83	354.09	226.23	175.08	218.58	68.06	176.66	82.32
After error correction by Trowel								
Min read length (bp)	10	9	6	9	13	13	12	8
Mean read length (bp)	57.99	57.99	57.98	57.98	55.99	55.99	55.99	55.97
Max read length (bp)	58	58	58	58	56	56	56	56
Inferred coverage	217.79	354.03	226.15	175.01	218.54	68.04	176.62	82.27

* Inferred coverage is calculated by (read length number of reads)/BEST195 genome length, where the BEST195 genome length is 4,105,380 bp. For corrected reads, we used the mean read length for calculations.

The result details for the analysis of "Insertion sequence"

Table B. BLASTn hits with the transposase of the insertion sequence in BEST195

	KorC1	LaoA1	MyaA2	ThaB	NepD5	NAFM5	NARUSE	TAKAHASHI
Total hit with IS	24	1	13	43	25	13	14	12
No. hit with IS4Bsu1	1	0	3	2	2	3	3	3
No. hit with ISBma2	1	1	3	1	1	2	1	1
No. hit with IS643	3	0	2	2	2	2	2	2
No. hit with IS256Bsu1	1	0	1	1	2	2	1	1
No. hit with ISLmo1	15	0	1	34	15	1	4	2
No. hit with putative transposase	3	0	3	3	3	3	3	3
Aligned with BEST195	21	0	9	35	23	13	13	11

The details for “Mapping and variant calling”

Table C. Sequence mapping and variant calls

	KorC1	LaoA1	MyaA2	ThaB	NepD5	NAFM5	NARUSE	TAKAHASHI
Mapping rate (%)	88.12	78.23	89.42	79.31	95.66	99.16	94.85	92.66
Coverage	185.34	259.5	194.28	125.09	200.98	64.26	161.95	74.6
[SD]	[77.32]	[130.19]	[82.5]	[53.23]	[81.3]	[23.85]	[86.42]	[64.27]
No. SNPs	20375	40548	17092	17010	5129	3	43	40
No. Indels	554	943	470	494	142	0	1	5
No. variants with high-impact	270	395	206	244	78	0	1	4
No. variants with moderate-impact	4806	9154	4035	3963	1243	0	14	26
No. genes with high or moderate-impact variants	1416	2233	1147	1214	365	0	6	23

Table D. Gene Ontology term (at level2) counts for genes with variations predicted to have high and moderate impacts for each strain

	KorC1	LaoA1	MyaA1	ThaB	NepD5	NARUSE	TAKAHASHI
Reproduction	1	4	1	1	1	0	0
Metabolic process	999	1562	785	811	250	13	12
Cellular process	894	1450	701	756	230	10	18
Biological adhesion	1	2	0	1	0	0	0
Signaling	86	109	65	54	12	0	3
Multicellular organismal process	1	0	1	0	0	0	0
Developmental process	11	42	14	18	10	0	2
Locomotion	6	16	5	4	1	0	1
Single-organism process	823	1317	647	711	234	15	17
Positive regulation of biological process	3	3	4	2	1	0	0
Negative regulation of biological process	0	4	0	0	3	0	0
Regulation of biological process	163	235	132	113	34	0	4
Response to stimulus	145	226	116	114	32	0	6
Localization	277	439	208	274	80	2	4
Multi-organism process	3	9	4	6	4	0	0
Biological regulation	164	239	133	114	34	0	4
Cellular component organization or biogenesis	28	67	23	32	13	0	2

The result details for the analysis "Biotin auxortophy of *B. subtilis* natto "

Table E. Variations in the *bio* operon

gene	locus_tag	strain	position	ref	alt	impact	effect
bioB	BSNT_09440	KorC1	2852638	A	C	MODERATE	NON_SYNONYMOUS_CODING
		LaoA1	2852418	A	T	MODERATE	NON_SYNONYMOUS_CODING
			2852638	A	C	MODERATE	NON_SYNONYMOUS_CODING
		MyaA2	2852418	A	T	MODERATE	NON_SYNONYMOUS_CODING
			2852638	A	C	MODERATE	NON_SYNONYMOUS_CODING
		ThaB	2852346	C	T	MODERATE	NON_SYNONYMOUS_CODING
			2852418	A	T	MODERATE	NON_SYNONYMOUS_CODING
			2852638	A	C	MODERATE	NON_SYNONYMOUS_CODING
			2852844	C	T	MODERATE	NON_SYNONYMOUS_CODING
bioD	BSNT_09441	KorC1	2853868	C	T	MODERATE	NON_SYNONYMOUS_CODING
		LaoA1	2853275	T	C	MODERATE	NON_SYNONYMOUS_CODING
			2853868	C	T	MODERATE	NON_SYNONYMOUS_CODING
			2853880	T	C	MODERATE	NON_SYNONYMOUS_CODING
			2853881	T	G	MODERATE	NON_SYNONYMOUS_CODING
			2853896	G	T	MODERATE	NON_SYNONYMOUS_CODING
		MyaA2	2853275	T	C	MODERATE	NON_SYNONYMOUS_CODING
			2853361	AC	A	HIGH	FRAME_SHIFT
			2853868	C	T	MODERATE	NON_SYNONYMOUS_CODING
			2853880	T	C	MODERATE	NON_SYNONYMOUS_CODING
			2853881	T	G	MODERATE	NON_SYNONYMOUS_CODING
			2853896	G	T	MODERATE	NON_SYNONYMOUS_CODING
		ThaB	2853275	T	C	MODERATE	NON_SYNONYMOUS_CODING
			2853868	C	T	MODERATE	NON_SYNONYMOUS_CODING
			2853880	T	C	MODERATE	NON_SYNONYMOUS_CODING
			2853881	T	G	MODERATE	NON_SYNONYMOUS_CODING
			2853896	G	T	MODERATE	NON_SYNONYMOUS_CODING
BSNT_09442	BSNT_09442	KorC1	2853964	TATGCAG	T	MODERATE	CODON_DELETION
			2853971	C	G	MODERATE	NON_SYNONYMOUS_CODING
			2853997	T	G	MODERATE	NON_SYNONYMOUS_CODING
			2854006	C	G	MODERATE	NON_SYNONYMOUS_CODING
			2854020	A	G	MODERATE	NON_SYNONYMOUS_CODING

			2854062	C	T	MODERATE	NON_SYNONYMOUS_CODING
			2854144	G	A	MODERATE	NON_SYNONYMOUS_CODING
		LaoA1	2853997	T	G	MODERATE	NON_SYNONYMOUS_CODING
			2854006	C	G	MODERATE	NON_SYNONYMOUS_CODING
			2854019	C	T	MODERATE	NON_SYNONYMOUS_CODING
			2854020	A	G	MODERATE	NON_SYNONYMOUS_CODING
			2854098	T	A	MODERATE	NON_SYNONYMOUS_CODING
			2854132	T	G	MODERATE	NON_SYNONYMOUS_CODING
			2854229	G	C	MODERATE	NON_SYNONYMOUS_CODING
			2854288	T	A	MODERATE	NON_SYNONYMOUS_CODING
			2854392	T	C	MODERATE	NON_SYNONYMOUS_CODING
			2854515	A	G	MODERATE	NON_SYNONYMOUS_CODING
			2854810	T	C	MODERATE	NON_SYNONYMOUS_CODING
			2854993	T	C	MODERATE	NON_SYNONYMOUS_CODING
		MyaA2	2853997	T	G	MODERATE	NON_SYNONYMOUS_CODING
			2854006	C	G	MODERATE	NON_SYNONYMOUS_CODING
			2854020	A	G	MODERATE	NON_SYNONYMOUS_CODING
			2854098	T	A	MODERATE	NON_SYNONYMOUS_CODING
			2854132	T	G	MODERATE	NON_SYNONYMOUS_CODING
			2854209	T	A	MODERATE	NON_SYNONYMOUS_CODING
			2854515	A	G	MODERATE	NON_SYNONYMOUS_CODING
		ThaB	2853997	T	G	MODERATE	NON_SYNONYMOUS_CODING
			2854006	C	G	MODERATE	NON_SYNONYMOUS_CODING
			2854020	A	G	MODERATE	NON_SYNONYMOUS_CODING
			2854098	T	A	MODERATE	NON_SYNONYMOUS_CODING
			2854132	T	G	MODERATE	NON_SYNONYMOUS_CODING
			2854209	T	A	MODERATE	NON_SYNONYMOUS_CODING
bioA	BSNT_09443	KorC1	2855174	G	T	MODERATE	NON_SYNONYMOUS_CODING
			2855831	A	G	MODERATE	NON_SYNONYMOUS_CODING
		LaoA1	2855120	T	A	MODERATE	NON_SYNONYMOUS_CODING
			2855751	T	C	MODERATE	NON_SYNONYMOUS_CODING
			2855831	A	G	MODERATE	NON_SYNONYMOUS_CODING
			2856293	G	T	MODERATE	NON_SYNONYMOUS_CODING
		MyaA2	2855120	T	A	MODERATE	NON_SYNONYMOUS_CODING

			2855193	T	A	MODERATE	NON_SYNONYMOUS_CODING
			2855350	A	G	MODERATE	NON_SYNONYMOUS_CODING
			2855751	T	C	MODERATE	NON_SYNONYMOUS_CODING
			2855831	A	G	MODERATE	NON_SYNONYMOUS_CODING
			2856209	T	C	MODERATE	NON_SYNONYMOUS_CODING
			2856293	G	T	MODERATE	NON_SYNONYMOUS_CODING
		ThaB	2855831	A	G	MODERATE	NON_SYNONYMOUS_CODING
BSNT_09444	BSNT_09444	LaoA1	2856499	T	G	HIGH	STOP_LOST
			2856615	T	C	MODERATE	NON_SYNONYMOUS_CODING
			2856630	G	A	MODERATE	NON_SYNONYMOUS_CODING
			2856911	C	T	MODERATE	NON_SYNONYMOUS_CODING
			2856936	A	G	MODERATE	NON_SYNONYMOUS_CODING
			2856972	G	A	MODERATE	NON_SYNONYMOUS_CODING
		MyaA2	2856499	T	G	HIGH	STOP_LOST
			2856555	T	C	MODERATE	NON_SYNONYMOUS_CODING
			2856815	T	C	MODERATE	NON_SYNONYMOUS_CODING
			2856911	C	T	MODERATE	NON_SYNONYMOUS_CODING
			2856936	A	G	MODERATE	NON_SYNONYMOUS_CODING

The result details for the analysis “Production of subtilisin NAT”

Table F. Variations in *aprN* and neighboring genes

gene	locus_tag	strain	position	ref	alt	impact	effect
yhfJ	BSNT_07462	LaoA1	1100394	A	C	MODERATE	NON_SYNONYMOUS_CODING
yhfK	BSNT_07463	KorC1	1100674	G	A	MODERATE	NON_SYNONYMOUS_CODING
			1100842	A	G	MODERATE	NON_SYNONYMOUS_CODING
			1101045	G	T	MODERATE	NON_SYNONYMOUS_CODING
			1101058	A	G	MODERATE	NON_SYNONYMOUS_CODING
		LaoA1	1100576	A	G	MODERATE	NON_SYNONYMOUS_CODING
			1100842	A	G	MODERATE	NON_SYNONYMOUS_CODING
			1101045	G	T	MODERATE	NON_SYNONYMOUS_CODING
		MyaA2	1100674	G	A	MODERATE	NON_SYNONYMOUS_CODING
			1100842	A	G	MODERATE	NON_SYNONYMOUS_CODING
			1101045	G	T	MODERATE	NON_SYNONYMOUS_CODING
			1101058	A	G	MODERATE	NON_SYNONYMOUS_CODING
		ThaB	1100842	A	G	MODERATE	NON_SYNONYMOUS_CODING
yhfL	BSNT_07464	KorC1	1101376	T	C	MODERATE	NON_SYNONYMOUS_CODING
			1102034	G	A	MODERATE	NON_SYNONYMOUS_CODING
		LaoA1	1102034	G	A	MODERATE	NON_SYNONYMOUS_CODING
			1102099	A	T	MODERATE	NON_SYNONYMOUS_CODING
		MyaA2	1101376	T	C	MODERATE	NON_SYNONYMOUS_CODING
			1102034	G	A	MODERATE	NON_SYNONYMOUS_CODING
		ThaB	1101469	A	G	MODERATE	NON_SYNONYMOUS_CODING
			1101600	G	T	MODERATE	NON_SYNONYMOUS_CODING
yhfM	BSNT_07465	KorC1	1102936	G	A	MODERATE	NON_SYNONYMOUS_CODING
		MyaA2	1102957	C	T	MODERATE	NON_SYNONYMOUS_CODING
		ThaB	1102957	C	T	MODERATE	NON_SYNONYMOUS_CODING
		NepD5	1102957	C	T	MODERATE	NON_SYNONYMOUS_CODING
yhfN	BSNT_07466	LaoA1	1103547	G	T	MODERATE	NON_SYNONYMOUS_CODING
			1103575	G	A	MODERATE	NON_SYNONYMOUS_CODING
			1104009	T	A	MODERATE	NON_SYNONYMOUS_CODING
			1104650	C	T	MODERATE	NON_SYNONYMOUS_CODING
		ThaB	1103575	G	A	MODERATE	NON_SYNONYMOUS_CODING
			1103673	C	G	MODERATE	NON_SYNONYMOUS_CODING

			1103869	A	G	MODERATE	NON_SYNONYMOUS_CODING
			1104009	T	A	MODERATE	NON_SYNONYMOUS_CODING
			1104644	A	G	MODERATE	NON_SYNONYMOUS_CODING
			1104646	A	G	MODERATE	NON_SYNONYMOUS_CODING
aprN	BSNT_07467	KorC1	1104970	A	G	MODERATE	NON_SYNONYMOUS_CODING
			1105061	T	A	MODERATE	NON_SYNONYMOUS_CODING
			1105157	T	A	MODERATE	NON_SYNONYMOUS_CODING
		LaoA1	1104970	A	G	MODERATE	NON_SYNONYMOUS_CODING
			1105061	T	A	MODERATE	NON_SYNONYMOUS_CODING
			1105157	T	A	MODERATE	NON_SYNONYMOUS_CODING
		MyaA2	1104970	A	G	MODERATE	NON_SYNONYMOUS_CODING
			1105061	T	A	MODERATE	NON_SYNONYMOUS_CODING
			1105157	T	A	MODERATE	NON_SYNONYMOUS_CODING
		ThaB	1104769	T	C	MODERATE	NON_SYNONYMOUS_CODING
BSNT_07468	BSNT_07468	KorC1	1106185	T	G	MODERATE	NON_SYNONYMOUS_CODING
			1106210	T	A	MODERATE	NON_SYNONYMOUS_CODING
			1106233	G	A	HIGH	STOP_GAINED
			1106252	AATTCGATT	A	HIGH	FRAME_SHIFT
			1106278	AT	A	HIGH	FRAME_SHIFT
			1106302	G	A	MODERATE	NON_SYNONYMOUS_CODING
		LaoA1	1106185	T	G	MODERATE	NON_SYNONYMOUS_CODING
			1106210	T	A	MODERATE	NON_SYNONYMOUS_CODING
			1106233	G	A	HIGH	STOP_GAINED
			1106252	AATTCGATT	A	HIGH	FRAME_SHIFT
			1106278	AT	A	HIGH	FRAME_SHIFT
			1106302	G	A	MODERATE	NON_SYNONYMOUS_CODING
		MyaA2	1106185	T	G	MODERATE	NON_SYNONYMOUS_CODING
			1106210	T	A	MODERATE	NON_SYNONYMOUS_CODING
			1106233	G	A	HIGH	STOP_GAINED
			1106252	AATTCGATT	A	HIGH	FRAME_SHIFT
			1106278	AT	A	HIGH	FRAME_SHIFT
			1106286	A	G	MODERATE	NON_SYNONYMOUS_CODING
			1106302	G	A	MODERATE	NON_SYNONYMOUS_CODING
yhfO	BSNT_07469	KorC1	1106335	G	A	MODERATE	NON_SYNONYMOUS_CODING

			1106344	C	A	MODERATE	NON_SYNONYMOUS_CODING
			1106456	C	T	MODERATE	NON_SYNONYMOUS_CODING
			1106558	C	T	MODERATE	NON_SYNONYMOUS_CODING
			1106563	T	C	MODERATE	NON_SYNONYMOUS_CODING
			1106587	G	T	MODERATE	NON_SYNONYMOUS_CODING
			1106621	G	A	MODERATE	NON_SYNONYMOUS_CODING
			1106732	C	G	MODERATE	NON_SYNONYMOUS_CODING
		LaoA1	1106344	C	A	MODERATE	NON_SYNONYMOUS_CODING
			1106456	C	T	MODERATE	NON_SYNONYMOUS_CODING
			1106558	C	T	MODERATE	NON_SYNONYMOUS_CODING
			1106563	T	C	MODERATE	NON_SYNONYMOUS_CODING
			1106621	G	A	MODERATE	NON_SYNONYMOUS_CODING
		MyaA2	1106344	C	A	MODERATE	NON_SYNONYMOUS_CODING
			1106347	G	A	MODERATE	NON_SYNONYMOUS_CODING
			1106414	T	A	MODERATE	NON_SYNONYMOUS_CODING
			1106558	C	T	MODERATE	NON_SYNONYMOUS_CODING
			1106563	T	C	MODERATE	NON_SYNONYMOUS_CODING
			1106587	G	T	MODERATE	NON_SYNONYMOUS_CODING
			1106621	G	A	MODERATE	NON_SYNONYMOUS_CODING
			1106732	C	G	MODERATE	NON_SYNONYMOUS_CODING
yhfP	BSNT_07470	LaoA1	1107069	G	A	MODERATE	NON_SYNONYMOUS_CODING
			1107489	G	A	MODERATE	NON_SYNONYMOUS_CODING
BSNT_07471	BSNT_07471	KorC1	1108102	G	A	MODERATE	NON_SYNONYMOUS_CODING
			1108345	C	G	MODERATE	NON_SYNONYMOUS_CODING
			1108444	C	T	MODERATE	NON_SYNONYMOUS_CODING
			1108541	T	C	MODERATE	NON_SYNONYMOUS_CODING
			1108835	T	G	MODERATE	NON_SYNONYMOUS_CODING
		LaoA1	1108122	A	C	MODERATE	NON_SYNONYMOUS_CODING
			1108177	A	G	MODERATE	NON_SYNONYMOUS_CODING
			1108345	C	G	MODERATE	NON_SYNONYMOUS_CODING
			1108522	G	A	MODERATE	NON_SYNONYMOUS_CODING
			1108541	T	C	MODERATE	NON_SYNONYMOUS_CODING
			1108835	T	G	MODERATE	NON_SYNONYMOUS_CODING
		MyaA2	1108122	A	C	MODERATE	NON_SYNONYMOUS_CODING

			1108177	A	G	MODERATE	NON_SYNONYMOUS_CODING
			1108345	C	G	MODERATE	NON_SYNONYMOUS_CODING
			1108541	T	C	MODERATE	NON_SYNONYMOUS_CODING
			1108835	T	G	MODERATE	NON_SYNONYMOUS_CODING
yhfR	BSNT_07472	KorC1	1109042	A	T	MODERATE	NON_SYNONYMOUS_CODING

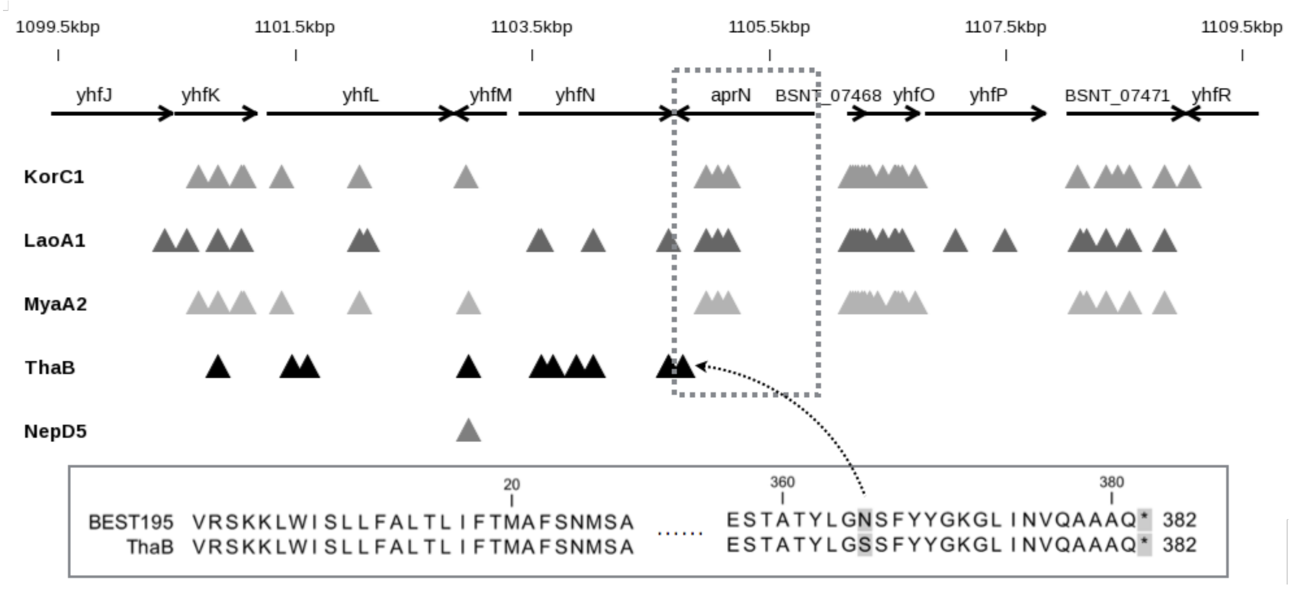

Figure A. The nucleotide changes in *aprN* and neighboring genes, and the amino acid sequence alignment in *Bacillus subtilis* BEST195 and strain ThaB. Triangle points represent a high or moderate variation at each position. A thymine-to-cytosine nucleotide change in *aprN* that leads to a non-synonymous coding mutation (asparagine to serine) was found only in strain ThaB.

The result details for the analysis “Production of γPGA ”

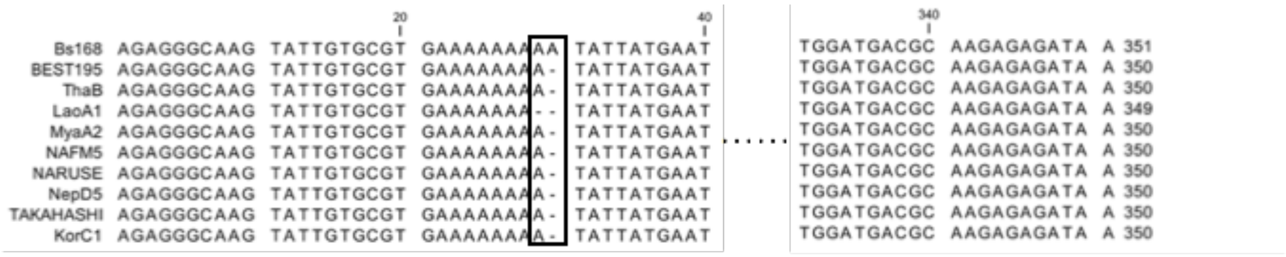

Figure B. The multiple sequence alignment of the eight *B. subtilis* strains, BEST195, and *B. subtilis* 168 at the corresponding region of *swrAA* (*yvzD* for BEST195). Every strain except strain LaoA1 has the same single adenine deletion that is present in BEST195.

Table G. Variations in genes constituting the ComQXPA quorum-sensing system

gene	locus_tag	strain	position	ref	alt	impact	effect
comA	BSNT_09631						
comP	BSNT_09632	KorC1	3015386	G	A	MODERATE	NON_SYNONYMOUS_CODING
			3015613	A	T	MODERATE	NON_SYNONYMOUS_CODING
			3015617	C	T	MODERATE	NON_SYNONYMOUS_CODING
		LaoA1	3015617	C	T	MODERATE	NON_SYNONYMOUS_CODING
		MyaA2	3015613	A	T	MODERATE	NON_SYNONYMOUS_CODING
			3015617	C	T	MODERATE	NON_SYNONYMOUS_CODING
comX	BSNT_09633						
comQ	BSNT_09634						

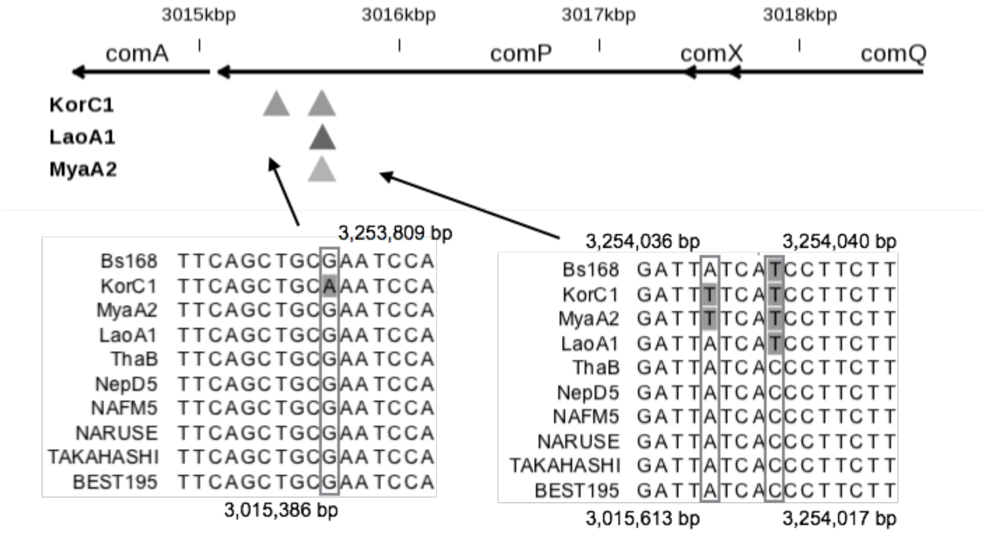

Figure C. The nucleotide changes in *comP* and the multiple sequence alignment of the corresponding regions. Triangle points represent a high or moderate variation at each position. A guanine-to-adenine nucleotide change was found only in strain KorC1, and an adenine-to-thymine nucleotide change was found only in strains KorC1 and MyaA2. Neither of the nucleotide changes were found in BEST195 and *B. subtilis* 168.

The result details for the analysis "Motility of *B. subtilis* "

Table H. Variations in genes constituting the bacterial flagellum

gene	locus_tag	strain	position	ref	alt	impact	effect
fliD	BSNT_10162	LaoA1	3495433	A	G	MODERATE	NON_SYNONYMOUS_CODING
			3495458	C	A	MODERATE	NON_SYNONYMOUS_CODING
			3495812	T	C	MODERATE	NON_SYNONYMOUS_CODING
			3495862	T	C	MODERATE	NON_SYNONYMOUS_CODING
			3495872	T	A	MODERATE	NON_SYNONYMOUS_CODING
			3495917	A	T	MODERATE	NON_SYNONYMOUS_CODING
			3495925	C	T	MODERATE	NON_SYNONYMOUS_CODING
			3495965	C	T	MODERATE	NON_SYNONYMOUS_CODING
			3496010	T	C	MODERATE	NON_SYNONYMOUS_CODING
			3496061	T	G	MODERATE	NON_SYNONYMOUS_CODING
			3496076	C	T	MODERATE	NON_SYNONYMOUS_CODING
			3496517	T	C	MODERATE	NON_SYNONYMOUS_CODING
			3496628	C	T	MODERATE	NON_SYNONYMOUS_CODING
			3496646	G	C	MODERATE	NON_SYNONYMOUS_CODING
			3496657	C	G	MODERATE	NON_SYNONYMOUS_CODING
			3496711	T	A	MODERATE	NON_SYNONYMOUS_CODING
			3496732	G	T	MODERATE	NON_SYNONYMOUS_CODING
			3496737	T	A	MODERATE	NON_SYNONYMOUS_CODING
			3496755	C	G	MODERATE	NON_SYNONYMOUS_CODING
			3496772	T	A	MODERATE	NON_SYNONYMOUS_CODING
			3496825	T	G	MODERATE	NON_SYNONYMOUS_CODING
			3496835	C	T	MODERATE	NON_SYNONYMOUS_CODING
flgL	BSNT_10168	LaoA1	3500119	T	C	MODERATE	NON_SYNONYMOUS_CODING
			3500441	C	A	MODERATE	NON_SYNONYMOUS_CODING
flgK	BSNT_10169	KorC1	3501144	A	T	MODERATE	NON_SYNONYMOUS_CODING
		LaoA1	3501144	A	T	MODERATE	NON_SYNONYMOUS_CODING
*BSNT_08139	BSNT_08139	LaoA1	1708952	A	G	MODERATE	NON_SYNONYMOUS_CODING
			1708977	G	T	MODERATE	NON_SYNONYMOUS_CODING
			1709116	A	G	MODERATE	NON_SYNONYMOUS_CODING
			1709178	G	A	MODERATE	NON_SYNONYMOUS_CODING
			1709203	A	G	MODERATE	NON_SYNONYMOUS_CODING

			1709219	G	A	MODERATE	NON_SYNONYMOUS_CODING
			1709244	T	A	MODERATE	NON_SYNONYMOUS_CODING
			1709263	G	A	MODERATE	NON_SYNONYMOUS_CODING
			1709275	GC	G	HIGH	FRAME_SHIFT
			1709279	T	TG	HIGH	FRAME_SHIFT
			1709282	G	A	MODERATE	NON_SYNONYMOUS_CODING
			1709297	C	T	MODERATE	NON_SYNONYMOUS_CODING
			1709346	T	G	MODERATE	NON_SYNONYMOUS_CODING
			1709364	G	T	MODERATE	NON_SYNONYMOUS_CODING
			1709365	C	G	MODERATE	NON_SYNONYMOUS_CODING
			1709424	T	G	MODERATE	NON_SYNONYMOUS_CODING
			1709521	A	C	MODERATE	NON_SYNONYMOUS_CODING
			1709765	T	C	MODERATE	NON_SYNONYMOUS_CODING
			1709848	T	C	MODERATE	NON_SYNONYMOUS_CODING
			1709911	A	G	MODERATE	NON_SYNONYMOUS_CODING
flgD	BSNT_08140	LaoA1	1710471	G	A	MODERATE	NON_SYNONYMOUS_CODING
			1710583	C	T	MODERATE	NON_SYNONYMOUS_CODING
flgG	BSNT_08141	LaoA1	1711102	A	C	MODERATE	NON_SYNONYMOUS_CODING
			1711234	G	C	MODERATE	NON_SYNONYMOUS_CODING
			1711349	A	C	MODERATE	NON_SYNONYMOUS_CODING
			1711422	G	T	MODERATE	NON_SYNONYMOUS_CODING
			1711431	T	G	MODERATE	NON_SYNONYMOUS_CODING
			1711432	C	T	MODERATE	NON_SYNONYMOUS_CODING
fliE	BSNT_08131						
fgB	BSNT_08129	ThaB	1702165	G	A	MODERATE	NON_SYNONYMOUS_CODING
flgC	BSNT_08130	LaoA1	1702495	A	G	MODERATE	NON_SYNONYMOUS_CODING
			1702589	T	A	MODERATE	NON_SYNONYMOUS_CODING
		ThaB	1702589	T	A	MODERATE	NON_SYNONYMOUS_CODING
motA	BSNT_07861	LaoA1	1449492	G	GCTT	MODERATE	CODON_INSERTION
			1450117	C	T	MODERATE	NON_SYNONYMOUS_CODING
motB	BSNT_07860						
*BSNT_08132	BSNT_08132	KorC1	1703577	T	TAATC TCCCG AAAG	HIGH	FRAME_SHIFT

		LaoA1	1703577	T	TAATC TCCCG AAAG	HIGH	FRAME_SHIFT
		MyaA2	1703577	T	TAATC TCCCG AAAG	HIGH	FRAME_SHIFT
		ThaB	1703305	A	G	MODERATE	NON_SYNONYMOUS_CODING
			1703561	A	G	MODERATE	NON_SYNONYMOUS_CODING
			1703582	T	G	MODERATE	NON_SYNONYMOUS_CODING
			1703597	G	A	MODERATE	NON_SYNONYMOUS_CODING
		NepD5	1703577	T	TAATC TCCCG AAAG	HIGH	FRAME_SHIFT
*BSNT_08133	BSNT_08133	LaoA1	1704272	A	T	MODERATE	NON_SYNONYMOUS_CODING
			1704423	A	G	MODERATE	NON_SYNONYMOUS_CODING
fliG	BSNT_08134						
fliM	BSNT_08144	LaoA1	1712703	A	T	MODERATE	NON_SYNONYMOUS_CODING
flhA	BSNT_08153	KorC1	1719233	T	A	MODERATE	NON_SYNONYMOUS_CODING
		LaoA1	1719233	T	A	MODERATE	NON_SYNONYMOUS_CODING
		MyaA2	1719233	T	A	MODERATE	NON_SYNONYMOUS_CODING
		ThaB	1719233	T	A	MODERATE	NON_SYNONYMOUS_CODING
			1719577	C	G	MODERATE	NON_SYNONYMOUS_CODING
flhB	BSNT_08152	LaoA1	1717483	A	G	MODERATE	NON_SYNONYMOUS_CODING
			1717697	G	A	MODERATE	NON_SYNONYMOUS_CODING
*BSNT_08135	BSNT_08135	LaoA1	1705840	T	C	MODERATE	NON_SYNONYMOUS_CODING
			1705945	G	A	MODERATE	NON_SYNONYMOUS_CODING
			1705966	A	G	MODERATE	NON_SYNONYMOUS_CODING
			1705987	A	G	MODERATE	NON_SYNONYMOUS_CODING
			1706030	C	T	MODERATE	NON_SYNONYMOUS_CODING
			1706032	T	A	MODERATE	NON_SYNONYMOUS_CODING
			1706035	C	A	MODERATE	NON_SYNONYMOUS_CODING
			1706069	G	C	MODERATE	NON_SYNONYMOUS_CODING
fliI	BSNT_08136						
fliJ	BSNT_08137						

fliP	BSNT_08148						
floQ	BSNT_08149						
*BSNT_08150	BSNT_08150						
fliT	BSNT_10160		3494769	C	T	MODERATE	NON_SYNONYMOUS_CODING
		LaoA1	3494829	C	T	MODERATE	NON_SYNONYMOUS_CODING
			3494897	T	G	MODERATE	NON_SYNONYMOUS_CODING
fliW	BSNT_10166		3498920	C	A	MODERATE	NON_SYNONYMOUS_CODING

* indicates gene annotations that have been changed in the BEST195 genome from the annotation in *B. subtilis 168*. *fliF* in *B. subtilis* 168 has been reannotated as BSNT_08132 and BSNT_08133 in BEST195. *fliK*, *filH*, and *filR* have been reannotated as BSNT_08139, BSNT_08135, and BSNT_08150 in BEST195, respectively.

The result details for MLST analysis

Table I. The identified allelic profiles for multilocus sequence typing (MLST) genes and the sequence type defined in PubMLST

	BEST195	168	KorC1	LaoA1	MyaA2	ThaB	NepD5	NAFM5	NARUSE	TAKAHASHI
glpF	3	1	61	3	1	3	3	3	3	3
ilvD	2	1	2	18	1	65	2	2	2	2
pta	2	1	2	2	2	4	2	2	2	2
purH	6	1	6	24	6	6	6	6	6	6
pycA	4	1	4	20	4	4	4	4	4	4
rpoD	3	1	3	1	3	1	3	3	3	3
tpiA	4	1	1	1	4	1	4	4	4	4
ST	4	1	-	11	-	-	4	4	4	4

ST: Sequence type defined in PubMLST based on the allelic profile. –, sequence type is not defined in the database.
